# Biotype Characterization, Developmental Profiling, Insecticide Response and Binding Property of *Bemisia tabaci* Chemosensory Proteins: Role of CSP in Insect Defense

**DOI:** 10.1371/journal.pone.0154706

**Published:** 2016-05-11

**Authors:** Guoxia Liu, Hongmei Ma, Hongyan Xie, Ning Xuan, Xia Guo, Zhongxue Fan, Balaji Rajashekar, Philippe Arnaud, Bernard Offmann, Jean-François Picimbon

**Affiliations:** 1 Shandong Academy of Agricultural Sciences, Biotechnology Research Center, Jinan, China; 2 University of Tartu, Institute of Computer Science, 2 Liivi, Tartu, Estonia; 3 University of Nantes, Protein Engineering and Functionality Unit, UMR CNRS 6286, 2 La Houssinière, Nantes, France; Zhejiang University, CHINA

## Abstract

Chemosensory proteins (CSPs) are believed to play a key role in the chemosensory process in insects. Sequencing genomic DNA and RNA encoding CSP1, CSP2 and CSP3 in the sweet potato whitefly *Bemisia tabaci* showed strong variation between B and Q biotypes. Analyzing CSP-RNA levels showed not only biotype, but also age and developmental stage-specific expression. Interestingly, applying neonicotinoid thiamethoxam insecticide using twenty-five different dose/time treatments in B and Q young adults showed that *Bemisia* CSP1, CSP2 and CSP3 were also differentially regulated over insecticide exposure. In our study one of the adult-specific gene (CSP1) was shown to be significantly up-regulated by the insecticide in Q, the most highly resistant form of *B*. *tabaci*. Correlatively, competitive binding assays using tryptophan fluorescence spectroscopy and molecular docking demonstrated that CSP1 protein preferentially bound to linoleic acid, while CSP2 and CSP3 proteins rather associated to another completely different type of chemical, i.e. α-pentyl-cinnamaldehyde (jasminaldehyde). This might indicate that some CSPs in whiteflies are crucial to facilitate the transport of fatty acids thus regulating some metabolic pathways of the insect immune response, while some others are tuned to much more volatile chemicals known not only for their pleasant odor scent, but also for their potent toxic insecticide activity.

## Introduction

Whiteflies are well known as severe agricultural pests, devastating all sorts of green ornamental and vegetable plants around the world. Whitefly species such as the sweet potato *Bemisia tabaci* Gennadius pierce and suck the sap, causing direct and indirect damages to various plant species. *Bemisia* damages the plants at all various moments of their life cycle. Females lay eggs on the inner surface of young leaves. Eggs will give birth to nymphs and adults that feed on the original leaf, releasing a sweet liquid (honeydew), which makes both the leaf and the fruit very sticky. Then a black fungus develops (fumago) and ultimately alters leaf photosynthesis and fruit cosmetic value. Among more than twenty bioforms, the biotype B of *B*. *tabaci* is known to cause squash silverleaf; this is how it received its most common name, “silverleaf whitefly” [1]. B expansion now worryingly shifts to another *Bemisia* biotype (Q), which turns out to become even more and extremely invasive throughout the whole world [2].

Hence, multiple studies have been initiated in *Bemisia* to understand better the biology of the different biotypes. The B and Q-biotypes of *B*. *tabaci* have the same development pattern, but are rather different in many other aspects including bacterial endosymbiont composition, dispersal behavior, fecundity, insecticide resistance and plant-host preference, among others [3]. They are morphologically indistinguishable share all common characteristics of the developmental processes from eggs to adults through four nymphal instar stages that are usually parasitized by wasps [4–5]. The mechanisms of defense in Q whiteflies have received immediate attention particularly since the biotype is known to develop a strong resistance to a high and large variety of insecticides, including mainly the neonicotinoids [6–10]. Still, we know so far very little about complete molecular mechanisms involved in this process.

Meanwhile, a large protein family called Chemo-Sensory Proteins (CSPs) has been described in various physiological systems of insects including Hemiptera [11–16]. CSPs have been originally described in regenerating legs of the cockroach *Periplaneta americana* and mature olfactory organs of *Drosophila melanogaster*, suggesting a function in development and olfaction depending on species [17–20]. CSPs have been proposed to play a role in olfaction as a sort of odor binding protein by delivering hydrophobic sensory molecules to sensory neurons in locusts and ants [21–22]. However, a role in relation with development has been seriously brought up in bees following the result of CSP knock out experiments. In *Apis mellifera*, the lack of functional CSP has been shown to cause abnormal head development [23]. This is in agreement with the occurrence of CSPs during various stages of the insect development as found in moths [24–25]. This is also in agreement with the very broad tissue expression profile characteristic of this very peculiar protein family [26–32].

In addition to olfaction and development, numerous other physiological functions have been suggested by various studies of the CSP family. The fact that a CSP protein (Mp10) is produced by the saliva of the green peach aphid *Myzus persicae* and triggers plant physiological defenses has suggested a role for CSP as effector protein rather than in the transportation of hydrophobic odorant chemical molecules [33]. Other similarly fancy experiments in moths have suggested a role of CSP as wetting agent to reduce the surface tension of aqueous sugar solutions and thereby the pressure involved in sucking nectar [34]. In contrast, the observation that expression of two CSP genes is up regulated during the response to bacterial infection in *D*. *melanogaster* has suggested some immunological function for this protein family [35]. In agreement with this observation, we have shown in a totally independent study that fourteen CSP genes in the silkworm moth *Bombyx mori* are significantly up regulated in various non-chemosensory tissues in response to insecticide avermectins [36]. These two studies together from *Bombyx* to *Drosophila* are in strong agreement with a role of CSPs in xenobiotic degradation and insect defense [35, 36].

In whiteflies, we previously reported a typical behavior of *B*. *tabaci* chemosensory protein-1 (BtabCSP1) in response to high dose of neonicotinoid Thiamethoxam [37]. We also reported genetic variation between B and Q biotypes for BtabCSP2, suggesting the occurrence of biotype-specific CSP mechanisms within aleyrods [38]. These two studies in *Bemisia* opened the question of what is the function of biotype-specific CSP in insecticide resistance, whether all CSPs from aleyrods have a role in relation with insecticide chemicals, what is the mode of action for those who have the ability to counteract insecticide, and what is the mode of action of others. Here, we described genes and RNAs encoding three different chemosensory proteins in the B and Q biotypes of the aleyrodid whitefly *B*. *tabaci* (*BtabCSP1*, *BtabCSP2* and *BtabCSP3*). We linked these three *Bemisia CSPs* with other functionally characterized *CSPs* from other insects and tested their behavior during the insect development and in response to different time-doses of insecticide thiamethoxam. We found in B and Q that *CSP1* and *CSP2* mainly expressed in adults, while *CSP3* expressed in third-instar nymphs. We then observed an extreme fluctuation of the *BtabCSP1* gene expression over time points after insecticide treatments specifically in biotype Q, while *CSP2* and *CSP3* expression levels did not change very significantly. Curiously, however, *BtabCSP2* expression fluctuated over time when insects were exposed to the plant leaf; it switched on in B when it switched off in Q. We thus tried to link the *Bemisia CSPs* with the expression of possibly related genes. Finally, we attempted to find interaction of CSP1, CSP2 and CSP3 with a true volatile or non-volatile cognate chemical ligand.

## Results

### BtabCSP1, BtabCSP2 and BtabCSP3 characterize *Bemisia tabaci* Q and B biotypes

**Fig 1:**
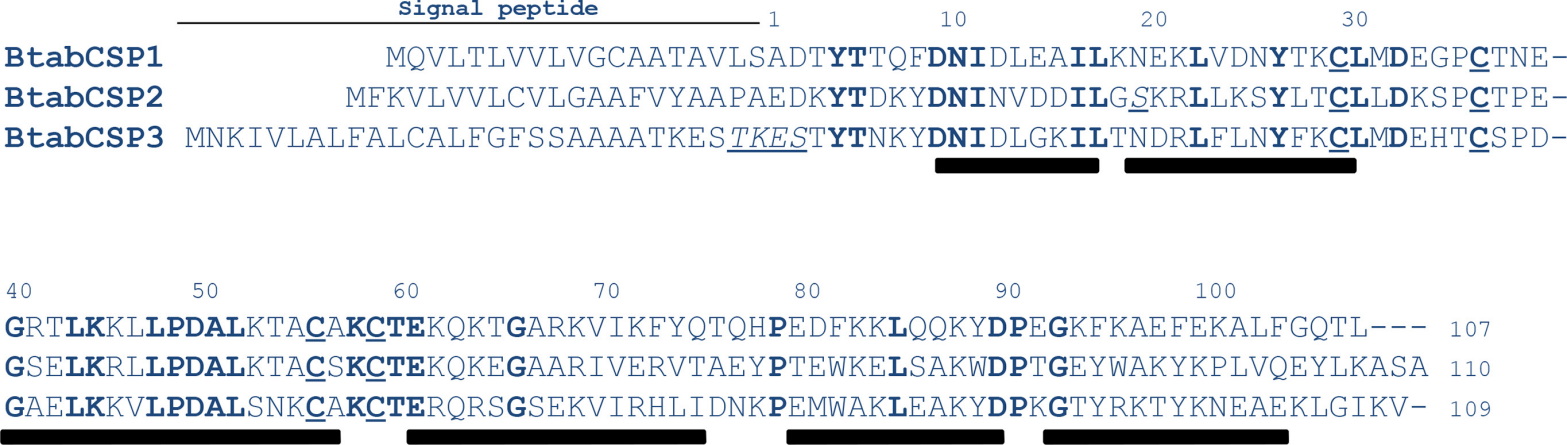
Identification of *B*. *tabaci* CSP1, CSP2 and CSP3. Conserved amino acids are shown in bold. The four Cys residues characteristic of CSPs are underlined. The residues or motifs in italic indicate specific mutations sites leading to amino acid replacement. Ala and Glu residues at position 1 are based on N-terminal sequencing of cockroach and moth CSPs [11, 13]. Bars in black indicate the position of putative alpha-helical domains (http://npsa-pbil.ibcp.fr).

Analyzing the EST database from the whitefly *B*.*tabaci* [4], three different sequences significantly related to CSPs were identified and called BtabCSP1, BtabCSP2 and BtabCSP3, respectively (Table 1). BtabCSP1, BtabCSP2 and BtabCSP3 are proteins of 107–110 amino acids with an isoelectric point of 6.61–8.84. Totally, thirty-four amino acids are strictly conserved between the three CSPs including the four cysteines characteristic of the CSP family. The middle part of the BtabCSP proteins (amino acid motif 40–70) is particularly well conserved. BtabCSP1, BtabCSP2 and BtabCSP3 rather differ in the N and C-terminal sequences (Fig 1).

**Table 1:** EST database of *B*. *tabaci* CSP clones.

Clone	NCBI	Contig sequence name	EST sequence name
BtabCSP1	KM078680-	BT-TYLCV-053-1-E12-T3_E12	BT-H-019-1-B4-T3_B04
	KM078685		BT-H-039-1-A4-T3_A04
			BT-TYLCV-053-1-E12-T3
			BT-TYLCV-053-1-B3-T3
		BT-TYLCV-059-1-A12-T3_A12	BT-TOMOV-027-1-F12-T3_
			BT-TYLCV-063-1-B8-T3_B08
			BT-H-031-1-A7-T3_A07
			BT-TOMOV-020-1-F4-T3_F04
BtabCSP2	KM078672-	BT-TYLCV-059-1-A12-T3_A12	BT_TYLCV002_A05
	KM078679		BT-TOMOV-043-1-A8-T3_A08
	KM078686-		BT-TYLCV-059-1-A12-T3_A12
	KM078691		HBT006_D09_T3_077
BtabCSP3	KM078692-	TOMOV-BT008_B11	BT-HINST-006-1-C12-T3_C12
	KM078697		BT-HINST-006-1-D2-T3_D02
			BT-HINST-010-1-H8-T3_H08
			BT-HINST-015-1-D9-T3_D09
			BT-HINST-016-1-H4-T3_H04
			BT-HINST-023-1-F6-T3_F06

The hydropathy plots of the three proteins are also very similar, showing high levels of hydrophobicity. However, the relative flexibility plots are rather different. Two flexible domains are predicted to occur in the protein BtabCSP1. The flexible domain in BtabCSP2 and BtabCSP3 is predicted to span over different regions (S1A Fig). The protein structure homology models built using MbraCSPA6 as template are very similar, except for C-terminal α-helix (S1B Fig). This was seen as a first indication that BtabCSP1 have different functions than CSP2 and CSP3.

We previously identified numerous substitution sites on the RNA encoding CSP1 in the B and Q biotypes of *B*. *tabaci* [37]. In this study, we found a high degree of RNA variance for CSP2, but not for CSP3 (S2 Fig). Some of these substitution sites in CSP2-RNA sequences (KM078671-KM078679) led to crucial changes in amino acid sequences, particularly in the motif between N-terminal α1 and α2-helices (Figs 1 and S1 and S1 and S2 Tables). However, CSP2 clones mostly differed by the C-terminal tail. In clones from B and Q (I74 and I80), an early stop codon led to the truncation of the three last residues ASA (S2 Fig and S1 and S2 Tables). Comparatively, only a few nucleotide replacements occurred on the RNA encoding CSP3 (KM078694-KM078697) although we cloned a mutation (I62-BtabCSP3) in Q that repeated three times the motif TKES in the N-terminal region (S2 Fig and S2 Table).

**Fig 2:**
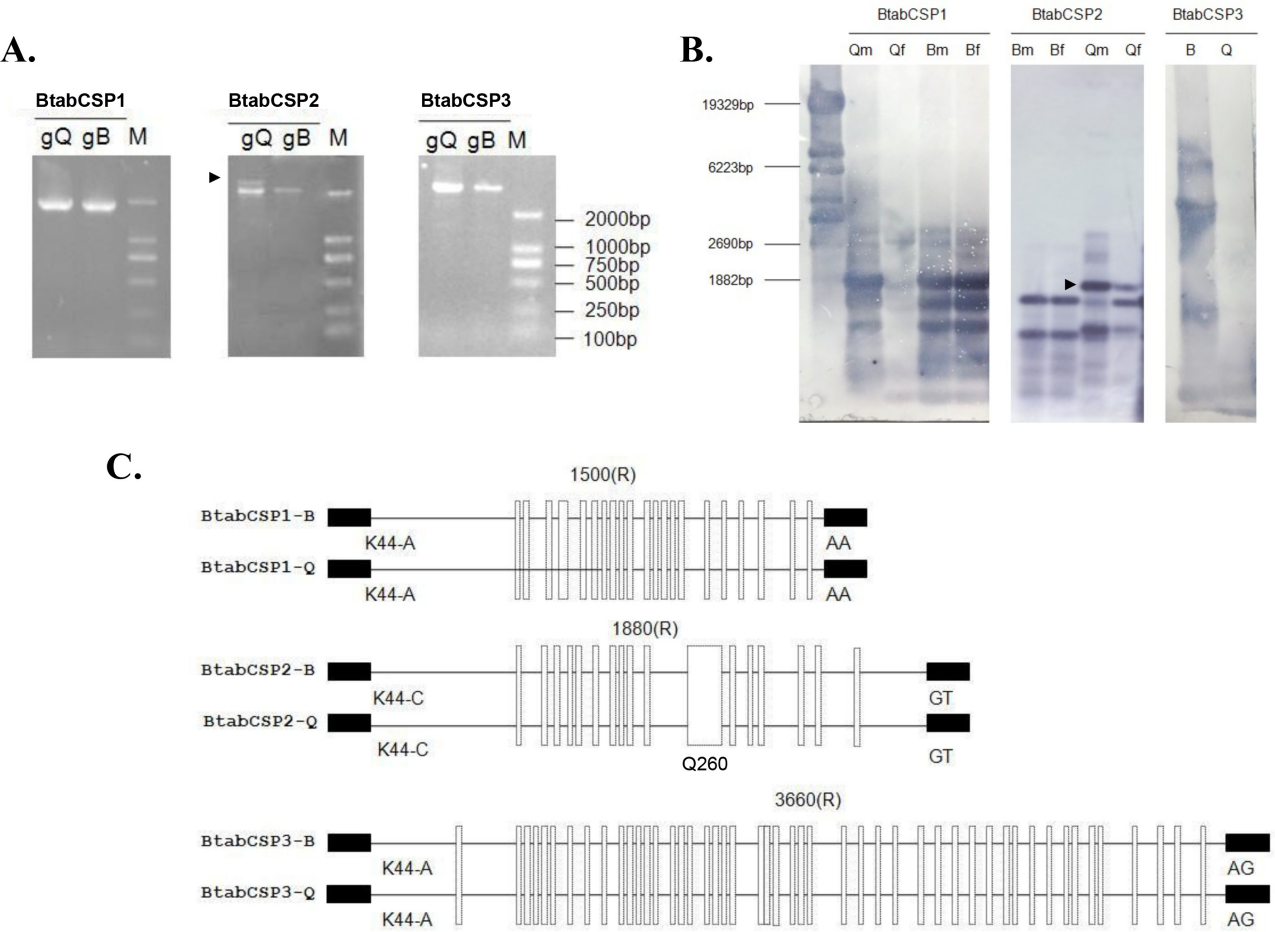
Identification of *B*. *tabaci* CSP1, CSP2 and CSP3 gene structures in B and Q biotypes. (A) Agarose gel electrophoretic analysis of genomic DNA PCR products from B and Q biotype using specific CSP1, CSP2 and CSP3 primers. (B) Southern blot analysis of genomic DNA PCR products isolated from *B*. *tabaci* in B and Q-biotype. Qm: Q males, Qf: Q females, Bm: B males; Bf: B females. A mixed pool of males and females were used for blot hybridized with CSP3 probe. Size markers (bps) are from HindIII-digested lambda DNA. The arrow tip shows CSP2-DNA band specific to Q. (C) Comparative analysis of gene structures encoding CSP1, CSP2 and CSP3 in B and Q biotypes of *B*. *tabaci*. Exon/intron boundaries (the first base of codon for K/R at position 45) were determined by comparing complementary and genomic DNA encoding CSP1, CSP2 and CSP3, respectively (KM078671-KM78697). Exons are represented by small black boxes, introns by straight black lines. The number above indicates the intron size. Mutation sites between B and Q for each CSP gene are shown by white vertical rectangles. Q260 indicates the position of intron motif specific to Q [38].

We thus aimed to identify the three CSP gene structures in selective PCR experiments. Using specific CSP1, CSP2 and CSP3 primers generated genomic DNA (gDNA) fragments of about 1800, 2000 and 5000 bps, respectively. Two gDNA fragments were amplified in Q for CSP2 gene (Fig 2A). Two 2 000 bps-gDNA bands for CSP2 were also detected in Q samples by Southern blot and DIG-labeling of PCR products. Gene diversity was observed also for *CSP1*. CSP1 probe hybridized to three main gDNA bands (Fig 2B). Using gDNA from ten individuals amplified repeatedly two CSP1 PCR bands for Q, suggesting that the number of CSP genes is different between Q and B biotypes of the sweet potato whitefly *B*. *tabaci* (S3 Fig). In contrast, PCR and blot using CSP3 probe identified only one major gDNA band of about 5 Kbs (Fig 2A and 2B).

Despite differences in the number of gene copies, sequencing gDNA encoding for CSP1, CSP2 and CSP3 in B and Q showed that the exon-intron structures were strictly conserved in the two biotypes. CSP1, CSP2 and CSP3 are single-intron genes of about 1500, 1880 and 3360 bps length, respectively (KM078680-KM078693). Intron differs in size, but it is always located at the same position (after the first base of codon for amino acid residue at position 45). Interestingly, we found widespread biotype B and Q sequence differences not only in gDNA encoding CSP1 and CSP2, but also in gDNA encoding CSP3 in the aleyrodid sweet potato whitefly *B*. *tabaci* (Fig 2C). The comparison between B and Q revealed a high number of sites where the gDNA sequence encoding for CSP1 from B differs from the corresponding gDNA sequence of the Q-biotype. The same observation was made comparing between B and Q the gDNA sequences encoding for CSP2 and CSP3, respectively (S4 Fig). Q even showed a very peculiar sequence (Q260) in the mid-region of the *CSP2* gene [38], demonstrating the occurrence of biotype-specific landmarks in *B*. *tabaci CSPs*.

### *BtabCSP1*, *BtabCSP2* and *BtabCSP3* represent three different orthology groups of *CSPs*

Analyzing amino acid sequences, a particularly high degree of similarity was found not only between whitefly and aphid CSPs, but also between CSPs from Hemiptera and various counterparts mainly from coleopteran, dipteran, lepidopteran and phthirapteran species (S5 Fig). *Bemisia* CSP1 was mainly related to aphid, fly and mosquito CSPs (about 50% identity, 70% similarity). *Bemisia* CSP2 found orthologs not only in aphids and flies, but also in beetles, mealy bugs and moths. *Bemisia* CSP3 was similar (about 70%) to CSPs from *Aedes*, *Culex*, *Ceratitis*, *Delia*, *Drosophila*, *Musca*, *Stomoxys* and many bark beetle, flour beetle, *Pediculus* and true bug species (S5 Fig).

**Fig 3:**
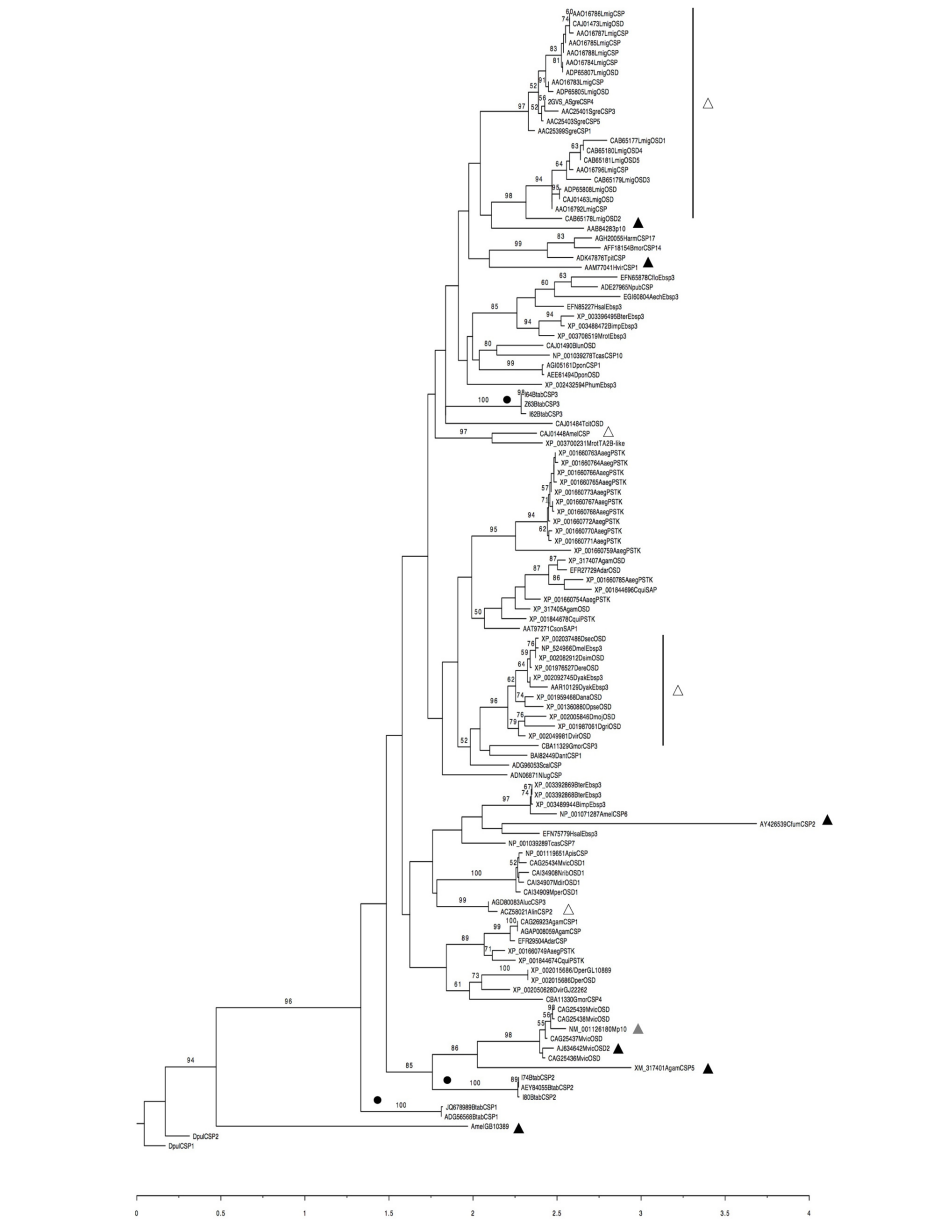
Phylogenetic analysis of amino acid sequences from *B*. *tabaci* CSP1, CSP2, CSP3 and orthologs in other insect species (RAxMLGUI). Aech: *Acromyrmex echinatior*, Apis: *Acyrthosiphon pisum*, Alin: *Adelphocoris lineolatus*, *Aaeg*: *Aedes aegypti*, Adar: *Anopheles darlingi*, Agam: *Anophales gambiae*, Amel: *Apis mellifera*, Aluc: *Apolygus lucorum*, Blun: *Biphyllus lunatus*, Bter: *Bombus terrestris*, Bimp: *Bombus impatiens*, Bmor: *Bombyx mori*, Cflo: *Camponotus floridanus*, Cson: *Culicoides sonorensis*, Dper/Dvir/Dyak: *Drosophila persimilis/virilis/yakuba*, Dpon: *Dendroctonus ponderosae*, Gmor: *Glossina morsitans morsitans*, Hsal: *Harpegnatus saltator*, Harm: *Heliothis armigera*, Lmig: *Locusta migratoria*, Mrot: *Megachile rotundata*, Mvic: *Megoura viciae*, Mdir: *Metopolophium dirhodum*, Mper: *Myzus persicae*, Npub: *Nylanderia pubens*, Nlug: *Nilaparvata lugens*, Nrib: *Nasonovia ribis nigri*, Phum: *Pediculus humanus corporis*, Sgre: *Schistocerca gregaria*, Scal: *Stomoxys calcitrans*, Tpit: *Thaumetopoea pityocampa*, Tcit: *Toxoptera citricida*, Tcas: *Tribolium castaneum*. Sequences are all published in NCBI. *B*. *tabaci* CSP1, CSP2 and CSP3 sequences are from JQ678989-ADG56568, KM078671- KM078679 and KM078694- KM078697, respectively. CSP sequences from *Daphnia pulex* (DpulCSP1 and DpulCSP2; ABH88167, ABH88166) are used as an outgroup. The triangles indicate the CSP proteins that have been functionally characterized. Black triangles: developmental genes, grey triangles: effector genes, white triangles: olfactory genes.

A phylogenetic analysis (RAxMLGUI) based on amino acid sequences of BtabCSP1, BtabCSP2, BtabCSP3 and CSPs from other insect species confirmed a clear relationship between BtabCSPs and CSPs from hemipteran and dipteran species (Figs 3 and S5). Using crustacean CSPs as outgroups, all of the CSPs used for the analysis fell on a common “insect” branch with high bootstrap values (94%). Interestingly, we noted that BtabCSP1 fell outside the main group of CSPs similarly to developmental *AmelGB10389* gene from honeybees, very distantly related to CSPs described in the olfactory system (Fig 3). In addition, on the phylogenetic amino acid tree, BtabCSP2 clearly grouped with effector protein Mp10 and developmental genes such as *AgamCSP5* and *MvicOSD*, suggesting that these four CSPs play a similar role eventually as effector proteins in both developmental and immunological systems (Fig 3). In contrast to BtabCSP1 and BtabCSP2, BtabCSP3 fell much closer to a large group including in particular locust olfactory CSPs/OSDs, the bee pheromone olfactory ASP3c gene (*CAJ01448*) and two known developmentally regulated CSP genes, p10 and HvirCSP1 (Fig 3). However, BtabCSP3 did not make a clear orthology group with any of the other *CSPs*. It even made a singleton despite close relatedness to TcitOSD (CAJ01484), a CSP from the brown citrus aphid, *Toxoptera citricida* (Fig 3). This strongly suggested a mechanism very specific to whiteflies for this peculiar type of CSP.

### *BtabCSP1*, *BtabCSP2* and *BtabCSP3* differentially express across various developmental stages of biotypes B and Q

We then compared the two biotypes B and Q of *B*. *tabaci* on expression of the three CSP genes identified (*BtabCSP1*, *BtabCSP2* and *BtabCSP3*).

Under control conditions, CSP1 and CSP2 showed preferential expression in biotype Q, while CSP3 gene expression was preferentially expressed in the biotype B. Slightly higher expression in males was detected for CSP1 and CSP2. No significant differences in CSP3 expression were found comparing males and females (S6 Fig).

**Fig 4:**
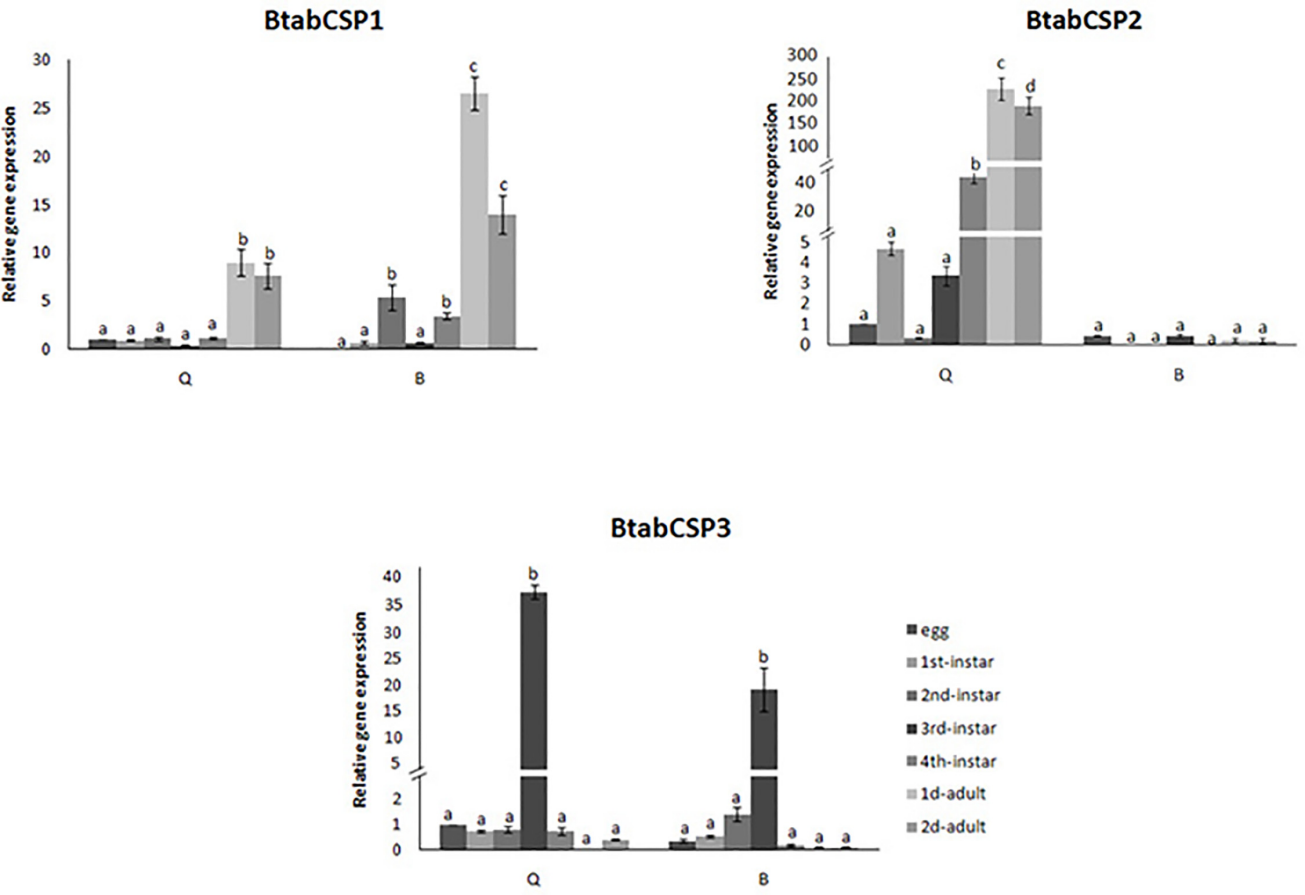
Differential expression of *B*. *tabaci* CSP1, CSP2 and CSP3 during development in biotypes B and Q. Relative gene expression levels for CSP1, CSP2 and CSP3 in B and Q biotype *B*. *tabaci* eggs, 1^st^, 2^nd^, 3^rd^ and 4^th^ instar nymphs and 1 to 2-days-old mixed adults (reference gene = β-actin). The relative expression levels observed in eggs were used for calibration (egg calibration value = 1). Data are means ± standard deviation (n = 9). Different letters indicate significant differences at α = 0.05 by one-way Anova. The symbol “═” on the Y-axis indicates very high expression of gene.

Detailed comparison of the gene expression profiles at seven developmental stages of the whitefly showed that CSP1 gene expression significantly increased by a factor of 10–30 at the adult stage in the two biotypes (Fig 4). CSP2 gene expression gradually increased starting at the third-instar stage to reach a 300-fold increase at the adult stage specifically in the biotype Q. Such an increase in CSP2 gene expression across the different developmental stages of *B*. *tabaci* was not observed in B biotype (Fig 4). In the two biotypes B and Q, CSP3 gene expression remarkably increased (by a factor of 20–40) in third-instar nymphs (Fig 4).

### *BtabCSP1*, *BtabCSP2* and *BtabCSP3* differentially express in response to age and thiamethoxam insecticide exposure

Hence, CSP1, CSP2 and CSP3 genes appeared to be differentially regulated across various developmental stages, with CSP1 and CSP2 highly expressed in adults, while CSP3 highly expressed in nymphs. This observation suggested different functions for *Bemisia* CSPs in a good agreement with such diversity in RNA variance, gene copy number and predicted structure.

We focused about a function in relation with insecticides and xenobiotics on the basis of the results from Sabatier et al. [35], Xuan et al. [36] and Liu et al. [37, 38].

**Fig 5:**
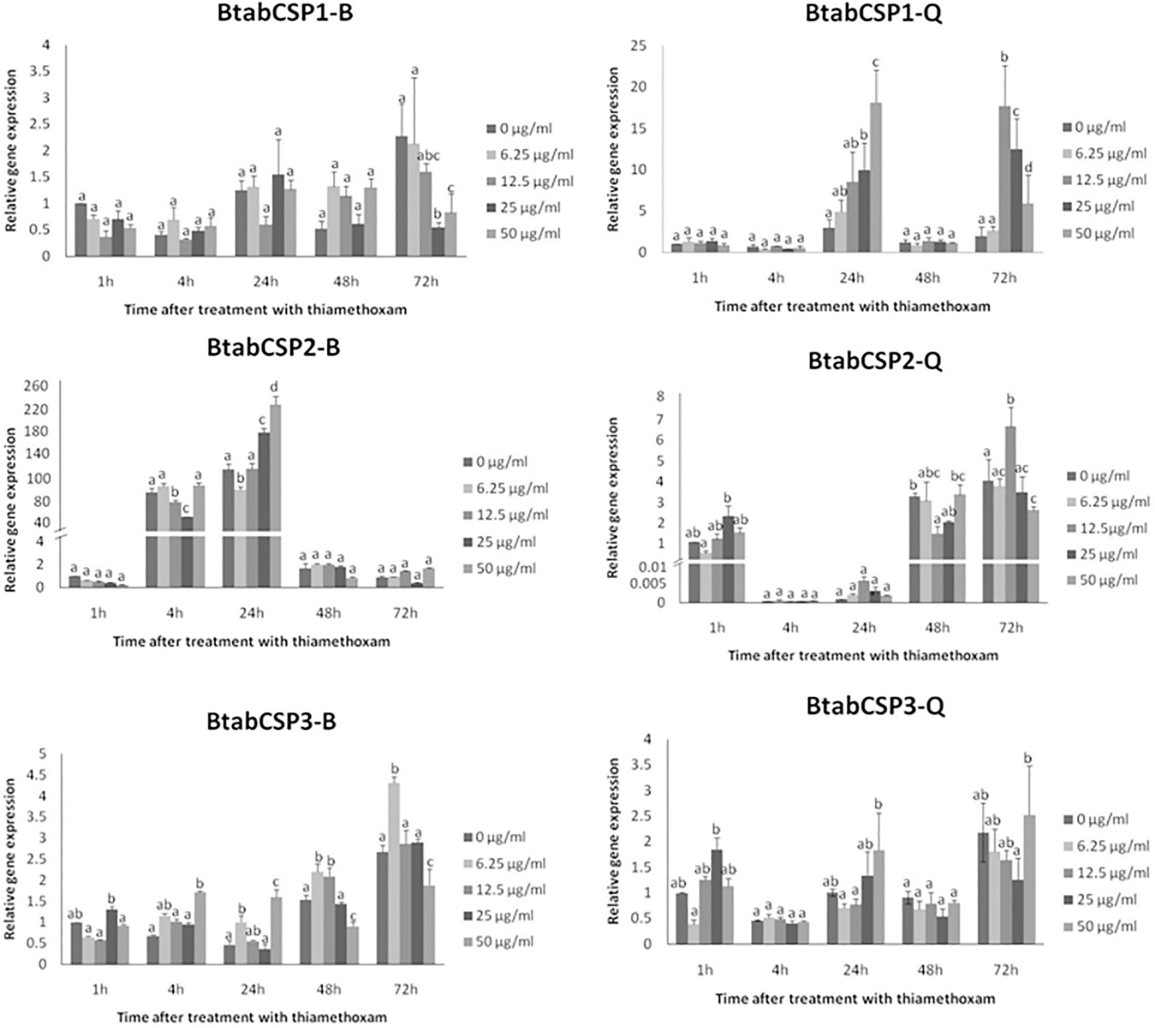
Modulation of *B*. *tabaci* CSP1, CSP2 and CSP3 expression over different time-dose treatments of thiamethoxam in biotypes B and Q. Relative gene expression levels for CSP1, CSP2 and CSP3 in B and Q biotype *B*. *tabaci* mixed adult individuals exposed to 0, 6.25, 12.5, 25 and 50 μg/ml dose of thiamethoxam (reference gene = β-actin). CSP1, CSP2 and CSP3 gene expression were measured in B and Q biotypes after 1, 4, 24, 48 and 72 h exposure. The relative expression levels observed in control individuals (0 μg/ml concentration of thiamethoxam) after 1 h were used for calibration (value = 1). Data are means ± standard deviation (n = 9). Different letters indicate significant differences at α = 0.05 by one-way Anova. The symbol “═” on the Y-axis indicates very high expression of gene.

Comparison of the gene expression profiles at five exposure times (1, 4, 24, 48 and 72 h) for five doses (0, 6.25, 12.5, 25 and 50 μg/ml) of insecticide thiamethoxam showed that CSP1 gene expression significantly increased in a time- and dose-dependent manner specifically in the biotype Q (Fig 5). After 1 and 4 h of insecticide treatment, no changes in CSP1 gene expression were noted for all five doses tested in the two biotypes. However, we observed in Q gradual increase in CSP1 gene expression using gradual concentrations of thiamethoxam after 24 h (Fig 5). No effects were seen in Q-whiteflies after 48 h, but stimulatory effects were observed after 72 h exposure to thiamethoxam dosed at 12.5 and 50 μg/ml, triggering an increase by a factor of about 10–25 in CSP1 gene expression (Fig 5). We did not observe such a time/dose effect of thiamethoxam on CSP1 expression in B (Fig 5). We observed no time/dose effect of thiamethoxam on CSP2 and CSP3 expression, neither in B nor Q (Fig 5).

However, we observed in the two biotypes an effect of age on CSP2 expression (100–200 folds increase), while no age-related effects were seen for CSP1 and CSP3 (Fig 5). Age-related effects on CSP2 were clearly opposite in B and Q; CSP2 up-regulated at 4 and 24 h in B, while it clearly up-regulated at 1, 48 and 72 h in Q (Fig 5), clearly illustrating biotype-specific changes with age in CSP2 expression in the sweet potato whitefly *B*. *tabaci*.

In order to understand the reason behind the modulation of *CSP1*, *CSP2* and *CSP3*, we subsequently analyzed gene expression for some possibly related genes such as *cytochrome P450 oxidase CYP6CM1* (GQ214539), *CYP6CX1* (GQ292715), *ecdysone receptor EcR* (EF174329), *trehalase* (JX024261) and *juvenile hormone binding protein JHBP* (see S7 Fig). We identified *B*. *tabaci* juvenile hormone binding protein (BtabJHBP) through the analysis of the whitefly EST database [4]. The EST clone BT-TYLCV-014-1-G6-T3_G06 showed between 30 and 60% identity with members of the JHBP superfamily from various insect species including mainly *Acyrthosiphon pisum* (XP_001949424) and *Reticulitermes flavipes* (ADM18966) in a blastx analysis (not shown). We saw that *BtabCSP3* and *BtabJHBP* had coordinate expression in nymphs (see Figs 4 and S7). In addition, we saw no correlation between *BtabCSP* expression and expression of *trehalase* and *EcR*, but we saw increased *CYP6CM1* expression in Q and reduced *CYP6CX1* expression in B in response to sublethal dose of insecticide thiamethoxam molecule as found for *CSP1* (see Figs 5 and S7) [37].

### BtabCSP1, BtabCSP2 and BtabCSP3 proteins differentially bind linoleic acid and cinnamaldehyde

**Fig 6:**
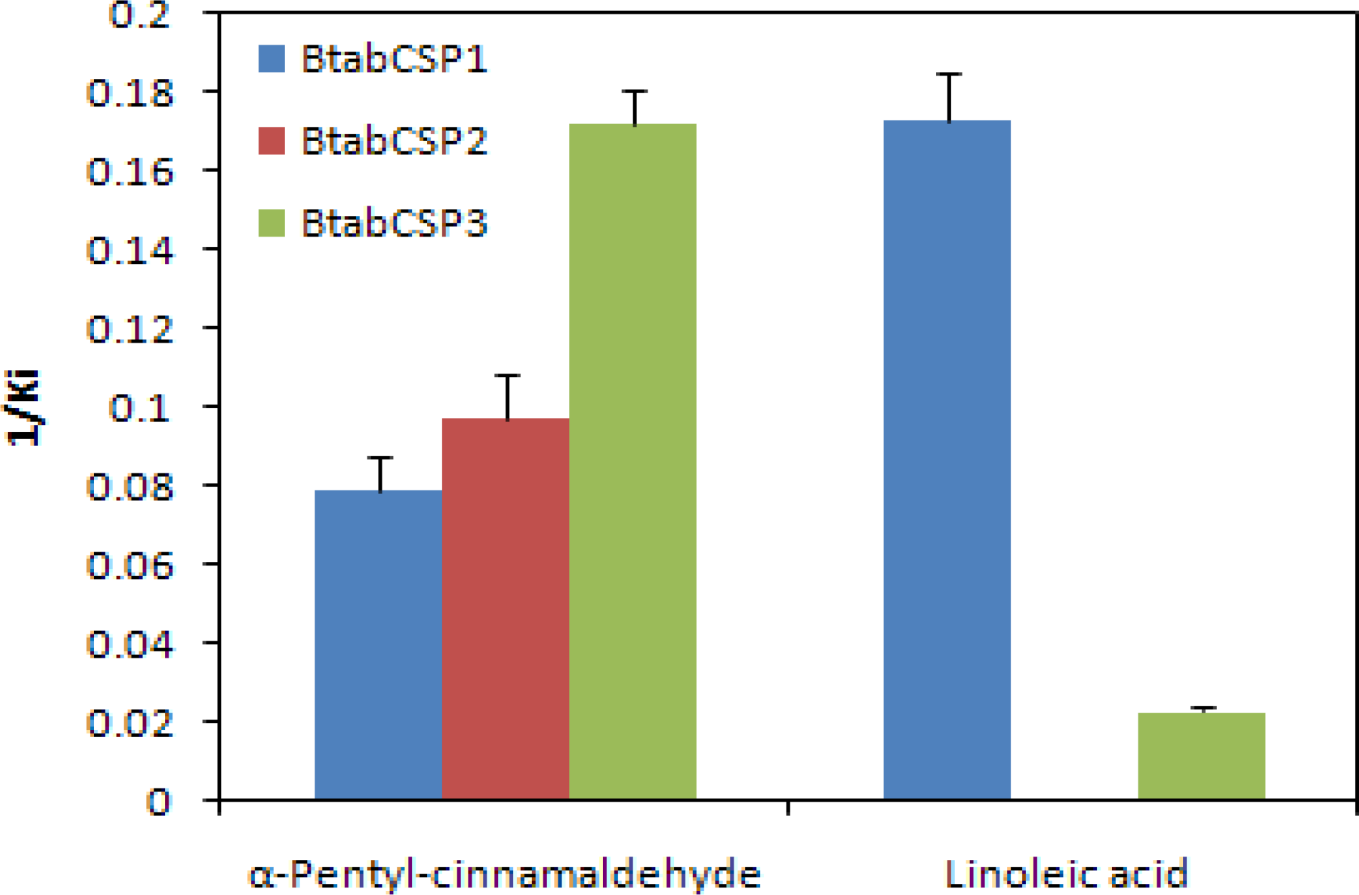
Fluorescence binding assay for the binding affinity of *B*. *tabaci* CSP1, CSP2 and CSP3. Binding constants (Ki as presented as 1/Ki) of BtabCSP1, BtabCSP2 and BtabCSP3 to linoleic acid (LA) and α-pentyl-cinnamaldehyde. The Ki values to various chemical ligands are given in Table 2. The highest binding constant value of BtabCSP1 (presented as 1/Ki) is found to LA (about 5.8 μM). Ki value of BtabCSP2 to LA is null, while Ki of BtabCSP3 to LA is only of about 48.5 μM. Higher binding constant values of BtabCSP2 and BtabCSP3 are to α-pentyl-cinnamaldehyde (5.8–10.3 μM).

**Fig 7:**
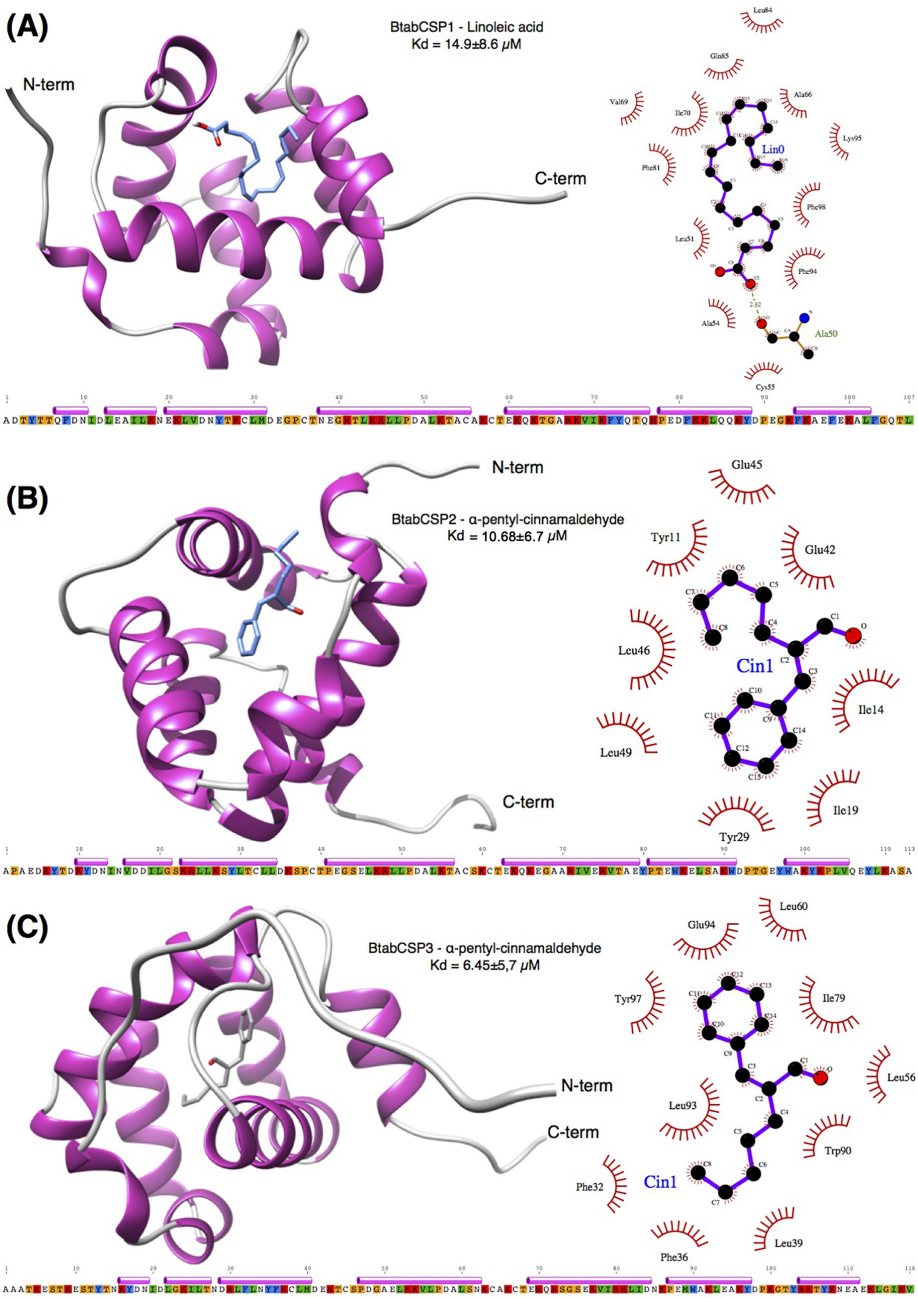
Docking analysis for the functional structure of *B*. *tabaci* CSP1, CSP2 and CSP3. Docking results showing (A) binding of linoleic acid to BtabCSP1, (B) binding of α-pentyl-cinnamaldehyde to BtabCSP2 and (C) binding of α-pentyl-cinnamaldehyde to BtabCSP3. Shown on the left are the cartoon representations of the bound ligands to the CSP models and on the right the Ligplot charts showing the residues that interact with the ligands. Amino acid sequences of mature peptides (after removal of peptide signal) and α-helical profiles for each CSP are provided below each cartoon representation. Residue numberings in these profiles are those that are featured in the LigPlot charts.

To complete our systemic investigation of CSPs of whitefly, we did a full comprehensive study analyzing not just CSP1, but also functions of CSP2 and CSP3 (Figs 6 and 7 and S8–S11, Tables 2 and 3 and S3 Table).

**Table 2:** Binding affinities of ligand chemicals to BtabCSP1, BtabCSP2 and BtabCSP3.

Ligand name	BtabCSP1 Ki (μM)	BtabCSP2 Ki (μM)	BtabCSP3 Ki (μM)	Ligand name	BtabCSP1 Ki (μM)	BtabCSP2 Ki (μM)	BtabCSP3 Ki (μM)
***Alcohols***				6-Methyl-5-hepten-2-one	u.d.	u.d.	u.d.
Trans-2-hexen-1-ol	46.12±1.25	u.d.	u.d.	2-Pentadecanone	u.d.	u.d.	u.d.
Linalool	u.d.	u.d.	u.d.	β-Ionone	32.10±1.48	u.d.	u.d.
1-Hexanol	46.85±1.37	u.d.	u.d.	2, 4′-Dimethylacetophenone	u.d.	u.d.	u.d.
Geraniol	39.63±3.01	u.d.	u.d.	***Carboxylic acids***			
Z3-Hexen-1-ol	u.d.	u.d.	u.d.	Octanoic acid	u.d.	u.d.	u.d.
(-)-Carveol	u.d.	u.d.	u.d.	Decanoic acid	u.d.	u.d.	u.d.
2-Ethyl-hexanol	u.d.	u.d.	u.d.	Linoleic acid	5.78±0.39	u.d.	43.48±2.86
3-Hexanol	u.d.	u.d.	u.d.	Cinnamic acid	75.46±2.29	u.d.	u.d.
α-Terpineol	u.d.	u.d.	u.d.	Lauric acid	35.22±1.42	u.d.	u.d.
(+/-)Nerolidol	u.d.	u.d.	u.d.	***Esters***			
Trans,trans-Farnesol	u.d.	u.d.	u.d.	Hexyl acetate	u.d.	u.d.	u.d.
1-Heptanol	u.d.	u.d.	u.d.	Amyl acetate	u.d.	u.d.	u.d.
***Aldehydes***				Methyl salicylate	60.01±2.74	u.d.	u.d.
Heptanal	u.d.	u.d.	u.d.	(Z)-3-Hexenyl acetate	u.d.	u.d.	u.d.
Trans-2-hexenal	u.d.	u.d.	u.d.	(E)-2-Hexenyl butyrate	u.d.	u.d.	u.d.
α-Pentyl-cinnamaldehyde	12.63±1.3	10.28±1.17	5.82±0.31	***Terpenes***			
Valeraldehyde	u.d.	u.d.	u.d.	Caryopyllene	u.d.	u.d.	u.d.
Nonanal	u.d.	u.d.	u.d.	α-Pinene	u.d.	u.d.	u.d.
Benzaldehyde	u.d.	u.d.	u.d.	β-Pinene	u.d.	u.d.	u.d.
n-Hexaldehyde	u.d.	u.d.	u.d.	Myrcene	u.d.	u.d.	u.d.
Octanal	u.d.	u.d.	u.d.	3-Carene	u.d.	u.d.	u.d.
Dodecyl aldehyde	u.d.	u.d.	u.d.	R(+)-Limonene	47.79±1.56	u.d.	u.d.
***Alkanes***				S(-)-Limonene	82.21±3.47	u.d.	u.d.
n-Undecane	u.d.	u.d.	u.d.	Ocimene	u.d.	u.d.	u.d.
n-Tridecane	u.d.	u.d.	u.d.	α-Terpinene	u.d.	u.d.	u.d.
n-Pentadecane	u.d.	u.d.	u.d.	E-β-Farnesene	u.d.	u.d.	u.d.
n-Hexane	u.d.	u.d.	u.d.	***Others***			
n-Heptane	u.d.	u.d.	u.d.	Eugenol	u.d.	u.d.	u.d.
***Ketones***				1,8-Cineole	u.d.	u.d.	u.d.
2-Heptanone	55.33±4.21	u.d.	u.d.	m-Xylene	55.51±3.14	u.d.	u.d.
(1S)-(-)-Camphor	50.83±3.08	u.d.	u.d.	Thiamethoxam	u.d.	u.d.	u.d.

u.d.: undetermined (binding constant was not calculated; IC_50_>100 μM)

Data are averages of three replicates (± SD).

**Table 3:** Molecular docking results for linoleic acid and α-pentyl-cinnamaldehyde on 3D models of BtabCSP1, BtabCSP2 and BtabCSP3.

Ligand	BtabCSP1(μM)	BtabCSP2 (μM)	BtabCSP3 (μM)
Linoleic acid	14.9±8.6	82±9.2	37.17±4.41
α-pentyl-cinnamaldehyde	12.36±4.73	10.68±6.7	6.45±5.7

We first used pure recombinant CSP protein samples, a selected repertoire of ligands and binding assay fluorescence spectroscopy (1-NPN) to analyze the functional properties of BtabCSP1, for which we found increased expression in Q following exposure to thiamethoxam (see Fig 5). In a preliminary binding assay, BtabCSP1 did not display certain binding ability with 25% pure thiamethoxam. Binding of thiamethoxam to CSP1 was found to be rather weak (IC50 = 326.26 μM, Ki = 285.43 μM), so there was not obvious relevance between insecticide binding to CSP protein and thiamethoxam induction (S9 Fig and Table 2).

To CSP1 protein, chemicals such as α-pentyl-cinnamaldehyde and S(-)-limonene showed a binding constant of 12.63±1.3 and 82.21±3.47 μM, respectively (Figs 6 and S9 and Table 2). The IC50 values obtained with other chemicals such as trans-2-hexen-1-ol, linalool, 1-hexanol, geraniol, Z3-hexen-1-ol, α-terpineol, nerolidol, n-hexaldehyde, n-heptane, 2-heptanone, (1S)-(-)-camphor, 6-methyl-5-hepten-2-one, β-ionone, octanoic acid, decanoic acid, cinnamic acid, lauric acid, amyl acetate, methyl salicylate, caryopyllene, myrcene, R(+)-limonene, ocimene, α-terpinene and m-xylene were comprised between 14.59±1.75 and 95.08±3.72 μM (Figs 6 and S9 and Table 2). No binding or IC50>100 μM was observed for other ligands including plant odor volatiles. However, in our selected set of 56 chemicals, we observed strong binding affinity of CSP1 to a eighteen carbons long fatty acid chain having three double bonds, i.e. linoleic acid (LA-CSP1: IC50 = 6.70±0.52, Ki = 5.78±0.39 μM; Figs 6 and S9, Table 2 and S3 Table).

We then set out to know the binding ability of BtabCSP2 and BtabCSP3 to most of all chemicals, but more precisely with linoleic acid and α-pentyl-cinnamaldehyde, respectively (Figs 6 and S8–S10 and Table 2). Importantly, no or low binding affinity of CSP2 and CSP3 was found for linoleic acid chemical (LA-CSP2: IC50 >100 μM; LA-CSP3: IC50 = 48.45±3.42, Ki = 43.48±2.86 μM; Figs 6 and S10 and Table 2). Even more importantly, both CSP2 and CSP3 proteins displayed significantly much higher binding affinity for the common direct contact toxic chemical of plant oil, α-pentyl-cinnamaldehyde (IC50 = 6.50–11.13±0.37–1.24, Ki = 5.82–10.28±0.31–1.17 μM; Figs 6 and S10, Table 2 and S3 Table), showing that CSP1 and CSP2 (and CSP3) from *B*. *tabaci* whiteflies have two completely different functions, although both related to detoxification and insect defense.

Finally, we studied the molecular structure of bound CSP1, CSP2 and CSP3 from *B*. *tabaci* using docking molecular techniques (Figs 7 and S11 and Table 3). Molecular modelling using multiple alignments that contained all seven CSP templates (1K19, 1KX8, 1KX9, 1N8U, 1N8V, 2JNT, 2GVS) failed to yield molecular models with the large cavity as observed in templates 1N8U and 1N8V. Molecular docking of linoleic acid and α-pentyl-cinnamaldehyde on these models gave binding modes where the ligands bound exclusively on the surface of the proteins with poor energies. Molecular modelling of BtabCSP1, BtabCSP2 and BtabCSP3 with the holo forms of the CSP as templates yielded models that displayed the presence of a large cavity that could accommodate linoleic acid and α-pentyl-cinnamaldehyde with much improved binding energies (Figs 7 and S11).

The docking showed that linoleic acid could be accommodated into the large cavity of BtabCSP1 after the ligand adopted a U shape (Fig 7). Indeed the aliphatic chain is too long to be accommodated in the cavity if it were to remain straight. The ligand establishes mainly hydrophobic contacts with cavity residues. Noteworthy, a hydrogen bond between the carboxylic group of linoleic acid and the C = O of the backbone of Ala50 was observed in this preferred binding mode (Fig 7). The predicted K_d_ value was 14.9±8.6 μM (Table 3). We observed several possible orientations of the same U-shaped linoleic acid in this large cavity with binding energy levels that were grossly equivalent. The same kind of binding modes were observed for linoleic acid in BtabCSP2 and BtabCSP3, but the predicted K_d_ values showed between three fold to six fold weaker binding affinities (Table 3).

Docking results for α-pentyl-cinnamaldehyde showed that all three CSPs could bind the ligand in the large cavity. The interactions are mainly hydrophobic, but clearly involved different sets of residues as shown for BtabCSP2 and BtabCSP3 in Fig 7. BtabCSP3 was predicted to have a higher affinity to the ligand with predicted Kd of 6.45±5.7 μM, which is about two fold higher than for BtabCSP2 and BtabCSP1 (Table 3).

## Discussion

We identified and analyzed the gene structures of three chemosensory proteins from the whitefly *Bemisia tabaci* (BtabCSPs). Four to twenty-one *CSPs* exist in the various insect species investigated so far [39–41]. Similarly, our PCR and Southern blot analysis of genomic DNA in the sweet potato whitefly *B*. *tabaci* is indicative of a high diversity in *CSPs* (undiscovered homologs, recently duplicated genes and/or pseudogenes). Therefore, *BtabCSP1*, *BtabCSP2* and *BtabCSP3* most probably do not represent the full complement of the CSP gene family in *Bemisia*. They represent, however, homologous genes sharing 37–43% identity with each other and probably the most abundant CSP-encoding RNAs expressed in the sequence tag database from adults and nymphs in *B*. *tabaci* (see Figs 1 and 2 and S1–S4 and Table 1) [4, 37, 38, 42].

Interestingly, the analysis of genomic DNA encoding CSP1, CSP2 and CSP3 allows us to clearly distinguish between the biotypes B and Q in the *Bemisia* complex (see Fig 2). The Q-biotype is known to have a higher adaptive capacity and in particular a stronger insecticide resistance capacity than the B-biotype [43–44]. Correlatively, we find that *BtabCSP1*, *BtabCSP2* and *BtabCSP3* are all three single-intron genes similarly to most of all *CSP*s, but clearly display biotype-specific hallmarks, particularly BtabCSP2 (see Figs 2 and S2 and S3) [38]. This suggests perhaps a link between the genetic repertoire of CSPs and the insecticide resistance capacities of the insect. Q-whiteflies retain a very specific genetic sequence (Q260) originating from bacteria that may be crucial for this process [38]. Furthermore, Q apparently contains more CSP genes than B (see Figs 2 and S3).

Also interestingly, we show a lot of biotype-specific variants of CSP1 and CSP2 transcripts (see S2 Fig and S1 and S2 Tables) [37]. This probably represents variation at the RNA level. A recent study from Xuan et al. in the silkworm moth *B*. *mori* have clearly demonstrated that variants of CSP transcripts do not represent variation at the genomic level, but rather tissue-specific RNA mutations resulting in the synthesis of multiple abundant CSP protein isoforms mainly differing by the terminal tails [45–46]. In addition, in the sweet potato whitefly *B*. *tabaci*, we show a high number of base substitution sites on the genomic DNA comparing CSP1, CSP2 and CSP3 between the two biotypes. We also show a different PCR profile analyzing CSP-encoding gDNA in Q and B individuals (see S3 Fig). Finally, we show that intron has been inserted or deleted in specific CSP genes during the evolutionary process of the two specific *Bemisia* biotypes (see S4 Fig). Confirming the results from Liu et al. [38], all these observations strongly indicate that B and Q *B*. *tabaci* biotypes are not truly unique species, subspecies or twin species but rather represent two very distinct species, each governed by very specific physiological systems [47–48].

Curiously, we find a lower degree of RNA and gDNA mutations for whitefly CSP3 compared to CSP1 and CSP2 (see S2–S4 Figs and S1 and S2 Tables). This is in agreement with the hypothesis that the degree of mutation depends on the function the protein has in a specific tissue of the insect [49]. A not striking but significant amino acid sequence similarity is found between these whitefly CSPs and some other CSPs from many various insect species (see Figs 3 and S5). A phylogenetic analysis based on amino acid sequences and implemented in RAxMLGUI shows insertion of BtabCSP1, BtabCSP2 and BtabCSP3 in a very large group of CSPs referring to many various functions. A BtabCSP1 ortholog in Hymenoptera (ASP3c, CAJ01448) found to be abundantly expressed in the antennae of adult honeybees and wasps is also found in high amounts in the hemolymph of drones [37, 50–52]. Another ortholog in bees (AmelGB10389) is involved in tegument development [23]. Orthologs in locusts are not only up-regulated during phase transition, but also highly expressed in antennae and legs at the adult stage [12, 53]. Other orthologs in flies are up-regulated in olfactory organs in adult alate swarmers [54–55], similarly to some orthologs from the alfalfa plant bug *Adelphocoris lineolatus* (AlinCSP2) [56]. Still, the same such CSP orthologs (pherokines) are found in hemolymph of virus-infected flies [35], suggesting a very general function for all of these CSPs. However, in our exhaustive study of two whitefly *B*. *tabaci* biotypes, we show at first glance that CSP1 and CSP2 preferentially express in Q, while CSP3 preferentially expresses in B (see S6 Fig), suggesting that at least these three CSPs in *Bemisia* might possibly be involved in some very different biotype-specific functions.

Interestingly, in our analysis of CSP functions in *B*. *tabaci*, we show that CSP1 and CSP2 are specific to the adult stage, suggesting a function in reproduction and/or plant odor detection at least for these two CSPs. In contrast, the extremely high levels of CSP3 expression at the third instar stage rather suggests a function in the preparation into adult stages, in particular in the development of sensory organs such as the antennae, head, eyes and various legs (see Fig 4). Many CSPs in other insect species have been shown to be associated with developing sensory organs [17, 23–25]. In addition, specifically in *B*. *tabaci*, a CSP from nymphs such as CSP3 could also play a function in wax lipid secretion and/or insect defense against parasitoids [57–58]. A function of CSPs in insect defense is strongly suggested by the analysis of genes abundantly expressed in the whitefly *B*. *tabaci* EST database from infected tissues [4]. No CSP-ESTs are found in the cDNA library of salivary glands in agreement with transcriptomic studies [59]. CSP3-ESTs are found in cDNAs from the instar library, but CSP1 and CSP2-ESTs are specifically found in the libraries of mature adults fed on tomato plants infected by the geminiviruses TYLCV (tomato yellow leaf curl virus) and TOMOV (tomato mottle virus) (see Table 1).

Hence, we set out to correlate the function of *Bemisia* CSPs to insect defense and in particular to whitefly resistance to neonicotinoid insecticide thiamethoxam. We measured transcript levels of CSP1, CSP2 and CSP3 genes with the treatments of five time points and five doses as well as the expression of genes involved in insecticide resistance [60]. The lifetime of adult whiteflies is of one month in the laboratory conditions. We have only used a population of young adults for our experiments, rejecting the possibility of biological mortal ratio of insects. There are probably variations due to various inter-individual physiological conditions, so we calibrated quantitative measurements of gene expression to a negative control (0 μg/ml) for each time point and for each CSP. The results confirmed previous data from Liu et al. [37].

There might be that CSP transcripts are regulated by many internal and external factors such as age, biotype, temperature and humidity, background odor, plant chemicals, light, circadian rhythm and season rhythm, etc., but for two time points, we show a clear dose effect of thiamethoxam on CSP1, particularly in biotype Q. CSP1 expression up-regulated 10–20 fold following exposure to sublethal doses of thiamethoxam at 24 and 72 h, but not at 48 h (see Fig 4). Perhaps it takes 48 h after involving CSP1 for full-system recovery to occur; perhaps it takes different durations of exposition to insecticide to recruit multiple resistance genes in a variety of defense mechanisms [61–62]. Time and dose dependent induction of detoxifying genes is usually observed at 24 h [63]. Hundreds genes are differentially expressed at 48 and 72 h post-infected *Bemisia* in response to fungal infection [64]. Similarly, we show only late responses to exposition of thiamethoxam molecule for CSP1 (see Fig 4). This delay could be due to the time needed for the thiamethoxam molecule to penetrate through the cuticle of adult whiteflies. However, it could also simply be that CSP1 is a late-response gene enrolled during prolonged exposure to xenobiotics. Sublethal doses of insecticide (6.25–50 μg/ml) are known to have variously delayed effects on key biological traits of the insect such as feeding behavior, honeydew excretion and fecundity [65–66]. Therefore, different CSPs could well be involved at different exposure time to protect various tissue-specific physiological pathways.

Nevertheless, in contrast to Q-CSP1, we note only little if any effects of thiamethoxam molecule on CSP2 and CSP3 from *Bemisia* (see Fig 4), suggesting that these two genes are not typically involved in the insect neonicotinoid response. BtabCSP2 and BtabCSP3 could be involved in the immunological response to another class of insecticides and/or simply have other functions than immunological defense.

It is noticeable in the sweetpotato whitefly *B*. *tabaci* that relative expression levels of *CSP1* and *CSP3* were stable over time in series of negative controls (0 μg/ml). Intriguingly, however, we found that *CSP2* was turned on in biotype B when it was turned off in biotype Q (see Fig 4). The phenomenon behind such an odd variation in CSP2 gene expression was rather mysterious. The vials were capped with an absorber cotton stopper, so there could not be an air contaminant or any signal released by B to inhibit Q. It could be instead a hormone or some toxic plant chemical that has differential effects on B and Q [67–68]. This was a very interesting point to characterize the CSP2 function by using same methods with CSP1 and CSP3.

In our study in *Bemisia* whiteflies we see some coordinate expression between CSPs and other gene families such as CYPs and JHBPs over development and/or insecticide exposure (see Figs 4 and 5 and S7). Coordinate expression of *CSP1* and *CYP6CM1* in *Bemisia* Q is in agreement with coordinate expression of *CSPs* and *CYPs* over insecticide exposure shown to be tissue-specific in *Bombyx* moths [36]. Two *CSPs*, *BmorCSP7* and *BmorCSP10*, and *JH esterase* had coordinate expression [36]. Taken together, the two studies suggest some relationships between CSP, CYP and JH related genes. The interactions among the CSP, CYP and JHBP genes probably need to be cautiously addressed by recombinant proteins interaction *in vitro*, RNAi, in situ hybridization and/or immunohistochemistry in moths, whiteflies and other insect species. It may be that some CSPs work with CYPs to mediate xenobiotics response, while some others play a key role in JH signaling pathways.

We have checked for the mode of action of BtabCSP1, BtabCSP2 and BtabCSP3 using protein expression, purification and fluorescence spectroscopic binding assay (see Figs 6 and S8–S10, Table 2 and S3 Table). Using BtabCSP1, for which we observed significantly increased expression in response to insecticide thiamethoxam molecule in Q biotype, we find no binding for volatile odorant molecules including terpenoids (see Figs 6 and S8 and S9, Table 2 and S3 Table), contrasting results obtained using various CSPs from mosquitoes and bugs [55, 69]. Using a similar binding assay, we find that BtabCSP1 rather involves in the binding of long fatty acid chains such as C18-linoleic acid as other proteins in the odorant binding protein and Niemann-Pick type C2 protein families [70–71]. The three 3D structures and binding data available for CSPs are in agreement with binding to various hydrophobic ligands including fatty acid molecules of various sizes and shapes [72–76]. Linoleic acid (delta 9,12 double bonds) is an unsaturated long carbon chain fatty acid molecule present in most of all insects in all tissues for lipid metabolism and fatty acid biosynthesis [77–78]. In the pheromone gland tissue, it is a key derivative of the fatty acyl intermediates from biosynthetic pathways for specific sex pheromone chemicals [79]. In addition, it is known to be involved in immunological response. Genes involved in linoleic acid metabolism are up regulated upon insecticide exposure along with CYP genes [80–81]. One function for linoleic acid is to help produce lipids and phospholipid-derived hormones that are directly involved in anti-xenobiotic response [82]. The molecule even can retain by itself an anti-xenobiotic activity [83]. Fatty acids such as linoleic acid molecule are therefore certainly crucial not only for various processes such as pheromone biosynthesis, hormone synthesis, neural sensory development and/or adult tissue growth, but also for immunological defense including general responses against insecticide or some other toxic xenobiotic chemicals.

Importantly, along our work on *Bemisia* CSP1 we show that two additional BtabCSPs do not bind fatty acid, but rather interact directly with specific toxic xenobiotic chemical. BtabCSP2 and BtabCSP3 proteins have a completely different functional task than CSP1. Using same binding assay method of CSP1, we clearly show that linoleic acid binds neither to CSP2 nor CSP3. CSP2 and CSP3 attach smaller, more volatile cyclic component structures such as α-pentyl-cinnamaldehyde with a much more higher affinity (see Figs 6, S8–S10, Table 2 and S3 Table). Interestingly, α-pentyl-cinnamaldehyde present in large amounts in plant essential oils is known not only for its pleasant cinnamon fragrance, but also for its very high insecticide activity [84–86]. This strongly suggests that, while CSP1s might be involved in the transport of fatty acids necessary to counteract neonicotinoid infection, CSP2s and CSP3s might rather be involved in the transport and direct degradation of much more volatile contact insecticide chemicals such as cinnamaldehydes and structurally related compounds.

We report molecular data in support of speciation and Q/B biotype-specific physiological pathways in the sweet potato/silver leaf whitefly *B*. *tabaci*. Our study shows that a particular CSP gene (BtabCSP1) expresses at the adult stage and responds to thiamethoxam intoxication specifically in the Q-biotype whitefly known to be highly resistant to neonicotinoid insecticides. Our study also shows that the BtabCSP1 gene encodes for a protein that binds particular long chain fatty acid, linoleic acid, among a large set of volatile and non-volatile ligands. The docking and 3D analysis of CSP1 with linoleic acid is supplied (see Figs 7 and S11 and Table 3), illustrating a profile of seven α-helices and detailed binding mechanism in the lipid solubilization process. BtabCSP1 bound to linoleic acid in a cavity formed by α3, α4 and α5 helices (see Figs 7 and S11). Residue numberings hereafter are as per the indications given in Figs 7 and S11. In the front of Cys55, linoleic acid molecule is sandwiched between Leu51-Ile 70-Phe81 and Ala66-Phe92-Lys95-Phe98 with the acid group anchored by Ala50 in the core of the protein. The extremity of the hydrophobic lipid chain is in close contact with the side chains of Phe81 and Phe98 and backbone of Lys95 (see Fig 7). This structural data is in good agreement with general distribution and behavior of most of all CSPs [20, 24–32, 36]. Particularly *CSP1* expression profiling strongly supports a general function in the transport of elongated lipids and fatty acids. In *Bemisia*, *CSP1* ubiquitously expressed in adult tissues from antennae to wing tips [20, 24–32, 36]. We note that *BtabCSP1* is particularly highly expressed in the wings (see S12 Fig). In the wings, fatty acids such as linoleic acid and proteins such as CSP1 may be able to determine the wettability and the ability to fly. In most other tissues including antennae and legs, the CSP-C18 complex may be essential for immunological response under xenobiotic stress such as exposure to insecticide (see Figs 5–7). Additionally, in most of all tissues, specific CSP-xenobiotic complexes are certainly crucial for specific responses of insect defense. While results from Sabatier et al. in the *Drosophila* fruit fly are in favor of an important role of CSPs in toxin degradation [35], our results in the *Bemisia* whitefly are in favor of a very important role of CSPs not only in the innate immune response, but also in adaptive (or acquired) immunity to a specific infectious agent such as a highly toxic host plant oil chemical molecule.

In this study, we also supply the docking and 3D analysis of CSP2 and CSP3 bound to cinnamaldehyde (see Fig 7 and S11 and Table 3). Similarly to CSP1, CSP2 and CSP3 are made of seven α-helices. The ring of the insecticide sits in the middle of Ile14-Ile19-Tyr29-Leu49 and Leu60-Ile76-Glu94-Tyr97 in CSP2 and CSP3, respectively. The aldehyde group interacts with Ile14 in CSP2, while it rather connects with Leu56 and Trp90 in CSP3. The extremity of the short aliphatic chain curves in the two proteins, folding in a net of amino acid residues including Tyr11-Glu42-Glu45-Leu46 (CSP2) and Phe32-Phe36-Leu39-Trp90-Leu93 (CSP3). BtabCSP2 and BtabCSP3 bound to the aromatic aldehyde in the central cavity of the prism, the N- and C-terminal α-helical tails remaining free (see Figs 7 and S11) [74]. This can be perhaps helpful to envision new strategies to interfere with fatty acid metabolism, cell signaling and/or insecticide resistance in an insect pest. Most of all BmorCSP genes are up regulated in the gut and other various tissues of the female silkworm infected by insecticide [36]. Our study presently shows that additional CSPs in *Bemisia* (BtabCSP2 and BtabCSP3) are able to directly interact with a strong potent contact insecticide volatile molecule, strongly suggesting the importance of CSPs at many different levels of the insect defense mechanism. It seems that this mechanism has reached an extremely high level of sophistication in insects, particularly in sweet potato whitefly *B*. *tabaci* biotype Q.

## Materials and Methods

### Ethics statement

*B*. *tabaci* B- and Q-biotypes were collected from eggplant fields in Shandong province (North/East-Penninsula, China). Shandong Academy of Agricultural Sciences gave permission to conduct field study on site (permit number SAAS 20110720). The field study did not involve endangered or protected species, but rather a very serious pest of agricultural crops worldwide.

### Insect rearing and tissue collection

Each *B*. *tabaci* biotype was identified using specific genetic markers, i.e. Cleaved Amplified Polymorphic Sequences (CAPs) of cytochrome oxidase I (*COI*) [87]. The two biotypes, B and Q, were then reared separately on mature cotton plants maintained in climatic incubators (26°C temperature, 60% humidity, 16/8 h day/night). Males were distinguished from females based on size and shape of the abdominal tip. The different development stages (crawlers, 2^nd^ to 4^th^ nymphs, pupae, adults) were distinguished microscopically based on size, shape, position of wings and color (red colored eyes). For each developmental stage present on the same infested plant, hundreds of individuals were collected in an eppendorf tube, kept on ice for 10 min and freeze-dried in nitrogen before storage at -70°C. Tissues were collected separately from a hundred of 1-2-days-old Q-adults under microscope and immediately frozen until RNA extraction.

### Molecular cloning of genomic DNA and cDNA

Antennal, abdominal, thoracic, leg, head and wing RNAs were extracted using Trizol for one-step Reverse Transcriptase PCR experiments. Whole body was used for RNA extraction in further molecular studies. Genomic DNA (gDNA) was extracted using DNeasy blood and tissue kit (Qiagen) for pools of thirty individuals in each biotype. Total RNA was extracted from the whole body of pooled individuals using the Trizol^TM^ method (Life Technologies) and treated with DNase I (Fermentas). Complementary DNA (cDNA) was synthesized using RevertAid^TM^ First Strand cDNA Synthesis Kit (Fermentas). Primers used to clone cDNA and gDNA of BtabCSP1, BtabCSP2 and BtabCSP3 were as follows:

BtabCSP1s: 5’-atgcaggttttgactttagtt-3’ (MQVLTLV),

BtabCSP1as: 5’-ttacagggtttgtccgaag-3’ (FGQTL),

BtabCSP2s: 5’-atgttcaaagttctcgtggt-3’ (MFKVLVV),

BtabCSP2as: 5’-ttaggcggatgccttgag-3’ (LKASA*),

BtabCSP3s: 5’-atgaacaagattgtattggc-3 (MNKIVLA),

BtabSCP3as: 5’-ctagactttgatacccaatt-3’ (KLGIKV*).

Additional primers tuned to specific intron fragments were used to sequence full-length gDNA clones:

CSP1-1f: 5’-GACTGTGGCAACCCTGAACGT-3’,

CSP1-1r: 5’-CGGTGAAATTAGATGTAGCCA-3’,

CSP2-1f: 5’-CAGAGGCCCAGACATTGCACC-3’,

CSP2-1r: 5’-GTAAATATCGGTGTTCTGA-3’,

CSP3-1f: 5’-TTTGGAAGAGTTTAGTGA-3’,

CSP3-1r: 5’-CTTGGCGCACTTGTTGGA-3’,

CSP3-2f: 5’-AGTCCACCCCAAAAACGATG-3’,

CSP3-2r: 5’-CTGGTTCTGGAAAAAGGTTGGT-3’,

CSP3-3f: 5’-AGGGTTGGTGCTTCTGAC-3’,

CSP3-4f: 5’-TTTAGAAAGACAAAGGACAA-3’.

PCR was done in a TaKaRa PCR Thermal Cycler Dice, programmed as follows: 94°C for 5 min; 35 cycles of 94°C for 30 s, 52°C for 30 s and 72°C for 1 min; final extension at 72°C for 7 min. PCR products were separated by electrophoresis in a 1% agarose gel stained with ethidium bromide. After staining, cDNA and gDNA PCR products of interest were purified using EasyPure Quick Gel Extraction Kit (TransGen Biotech) and cloned into pEasy T1 vector (TransGen Biotech). For each biotype-specific cDNA or gDNA PCR product, ten clones were subjected to sequencing on an ABI3700 sequencer instrument using the RR Dye Deoxy terminator cycle sequencing kit (Perkin Elmer) and Sp6/T7 sequencing primers.

### Southern blot

PCR products were amplified from specific BtabCSP1, BtabCSP2 and BtabCSP3 plasmids as templates, purified and labeled with Digoxigenin-11-dUTP using DIG-High Prime (DIG High Prime DNA Labeling and Detection Starter Kit I; Roche). Genomic DNA sequences corresponding to BtabCSP1, BtabCSP2 and BtabCSP3 were amplifed in biotypes B and Q using HiFi Taq (TransGen Biotech). PCR products and labeled λ-EcoR T14 digest DNA markers were separated using 0.8% agarose gel electrophoresis at low voltage for 10 h. After denaturation and neutralization, PCR products and markers were transferred from gel to nylon membrane by capillary transfer using 20× SSC for 20 h. Following transfer, nylon membrane was backed in a vacuum oven maintained at 80°C for 2 h. Membrane DNA was then prehybridized in DIG Easy Hyb buffer at 42°C for 2 h and hybridized with specific DIG BtabCSP probe in DIG Easy Hybridization buffer (12 rpm, 39°C, overnight). After hybridization, membrane was incubated in 2× SSC/0.1%SDS (2 × 5min) and stringency wash solution (0.5× SSC/0.1% SDS) at 65°C for 2 × 15min on a shaker agitator. Immunological detection was done according to DIG High Prime DNA Labeling and Detection Starter Kit I (Roche).

### Bioinformatic analysis

The 9110 Sequences from the EST whitefly database were downloaded from GenBank (www.ncbi.nlm.nih.gov) and analyzed using the blastx algorithm of the NCBI database. In this sequence similarity study, eighteen clones showed specific hits to OS-D superfamily (Table 1). Sequences orthologous to BtabCSP1, BtabCSP2 and BtabCSP3 in other insect species were searched by screening the NCBI database using the BLASTP tool (http://blast.ncbi.nlm.nih.gov/). Only the sequences having total score above 120, query coverage above 90%, e-value below 1e-33 and a maximum identity superior to 50% were selected for phylogenetic analysis.

For phylogenetic analysis, BtabCSP and orthologous sequences were aligned in MUSCLE Version 3.8.31. The resulting PHYLIP file (SEAVIEW; http://pbil.univ-lyon1.fr/software/seaview3) was used as template for further processing in RAxMLGUI [88]. In RAxMLGUI, the phylogenetic tree was built using parsimony with the inclusion of maximum likelihood (PROTGAMMA model, BLOSUM62, 100 boostraps). The two CSP sequences (ABH88167, ABH88166) from the water flea *Daphnia pulex* (Arthropoda, Crustacea, Branchiopoda, Cladocera, Daphniidae) were used as outgroup.

### Quantitative real-time PCR (qRT-PCR)

One-step Reverse transcriptase PCR (TAKARA Bio. Inc) was used to check for tissue-distribution of CSP1 following the method described in Liu et al. [37]. qRT-PCR was used to measure the changes of CSP1, CSP2 and CSP3 gene expression during development and over insecticide exposure. In a typical qRT-PCR experiment, cDNA templates (20 ng/μl) were used in a total volume of 20 μl containing SYBR Green I PCR Master Mix (Roche) and 200 nM of each primer, using 96-well optical-grade PCR plates and matched optical-grade membrane of StepOne Plus ABI system. qPCR primers were specifically designed to generate an amplicon of about 100–150 bps:

qPCR-BtabCSP1s: 5’-gacaactacaccaagtgcc-3’ (DNYTKC),

qPCR-BtabCSP1as: 5’-gtagaacttgatgaccttacg-3’ (RKVIKFY),

qPCR-BtabCSP2s: 5’-caacgtcgacgatattttgg-3’ (NVDDIL),

qPCR-BtabCSP2as: 5’-cttttctgtgcatttggagc-3’ (CSKCTE),

qPCR-BtabCSP3s: 5’-tgatggatgaacacacatgc-3’ (LMDEHTC),

qPCR-BtabCSP3as: 5’-gaatgaccttttcggatcca-3’ (SGSEKVIR).

We used actin (housekeeping gene) as an internal invariant endogenous control for qRT-PCR (BtabActin-sense: 5’-tcttccagccatccttcttg-3’, BtabActin-antisense: 5’-cggtgatttccttctgcatt-3’; BT-H-019-1-C5-T3_C05). We also compared in each body sample the expression values of CYP6M1, CYP6X1, JHBP, EcR, USP and trehalase genes referred to *BtabActin* as control (= 1). Primers were as follows (GQ214539, GQ292715, G6-T3_G06, EF174329, EF174330, JX024261):

qPCR-BtabCYP6CM1s: 5’-tccacgagggaattttagct-3’,

qPCR-BtabCYP6CM1as: 5’-gcagcgtctcatcaataactt-3’,

qPCR-BtabCYP6CX1V1s: 5’-gtgccctacatctcgcctatca-3’,

pPCR-BtabCYP6CX1V1as: 5’-ccagccactctttcaccattcc-3’,

pPCR-BtabEcRs: 5’-actcaacgacgagttgttctcc-3’,

pPCR-BtabEcRas: 5’-gctacattttcttcttctggtgc-3’,

pPCR-BtabTres: 5’-caacaatgggacgcacaaa-3’,

pPCR-BtabTreas: 5’-ctcacctccgcctccaatag-3’,

pPCR-BtabJHBPs: 5’-aacggaaaccaaagactgag-3’,

pPCR-BtabJHBPas: 5’-ggaacacttggttgagtaca-3’.

qRT-PCR products were sequenced to attest primer specificity. Each sample in B and Q was analyzed in triplicates. Three biological samples were used for each time point at each developmental stage and in the experiments on the effects of insecticide on the expression of CSP genes (see below). Reactions were run in a StepOne Plus system with initial denaturing step consisting of 2 min at 50°C and 10 min at 95°C, followed by 40 cycles of 15 s at 95°C-30 s at 60°C and terminated by a dissociation curve program from 60 to 95°C with a heating rate of 0.1°C and a continuous fluorescence acquisition. Expression levels were determined using the relative 2(-Delta Delta C(T)) method [89]. Data were statistically analyzed by one-way Anova in SPSS.

### Thiamethoxam assay

Thiamethoxam is one major neonicotinoid insecticide, known to act on the nicotinic acetylcholine receptors (nAChR) within the insect central nervous system. To expose *B*. *tabaci* to the neonicotinoid insecticide thiamethoxam (25% WG, Syngenta, China), we chose the cotton leaf-dip method [37, 90]. One week<-old adult forms from the laboratory colonies of B- and Q-biotypes of *B*. *tabaci* were collected separately and exposed in the laboratory conditions (26°C temperature, 60% humidity, 16/8 h day/night) to 0, 6.25, 12.5, 25 and 50 μg/ml of thiamethoxam, respectively. Significant mortality rate was observed only for higher doses (>100 μg/ml) at different exposure time. A 100 μg/ml dose of insecticide killed most of the whiteflies after 72 h exposure.

The experiment started (time 0) when insecticide treated and non-treated plant discs (2.5 cm diameter) were individually placed on agar base in 20-ml glass vials.Twenty-eight whiteflies belonging either to B or Q-biotype were aspirated into each vial. Vials were capped with an absorbent cotton stopper and inverted. Insecticide-treated whiteflies fed in treated vials on cotton plant leaf discs dipped for 10 s in formulated thiamethoxam diluted with distilled water. Whiteflies kept in vials containing cotton plant leaf discs of the same diameter dipped for 10 s in distilled water only were used as controls (0 μg/ml of thiamethoxam). After 1, 4, 24, 48 and 72 h post-exposure, all individuals in each category were collected in an eppendorf tube in treated and untreated groups of whiteflies in each of the two biotypes, kept on ice for 10 min and freeze-dried in nitrogen before storage at -70°C.

### Protein expression and purification

Recombinant CSP1, CSP2 and CSP3 proteins were expressed using Champion^TM^ pET SUMO protein expression system (Invitrogen) according to the Invitrogen protocol. The coding region was amplified by PCR using two specific primers:

BtabCSP1prots: 5’-GCCGATACCTACACGACCCA-3’,

BtabCSP1protas: 5’-TTACAGGGTTTGTCCGAAGAGG-3’.

BtabCSP2prots: 5’-GAAGACAAATACACGGACAAATATGA-3’

BtabCSP2protas: 5’-TTAGGCGGATGCCTTGAGGT-3’

BtabCSP3prots: 5’-GAAAGTACGTACACGAATAAATACCAC-3’

BtabCSP3protas: 5’-CTAGACTTTGATACCCAATTTTTCG-3’

For protein expression, specific PCR products were ligated into Pet SUMO vector, before to use the construct to transform competent One Shot Mach1-T1 *E*.*coli* cells. Sequencing a dozen of clones identified constructs with correct orientation and gene sequence. The CSP1, CSP2 and CSP3 constructs were all three sub-cloned into BL21 (DE3) One Shot cells. Single colonies were grown in 10 ml LB medium containing 50 μg/ml kanamycin at 37°C with shaking (200 rpm) overnight. The next day, 50 ml LB medium containing 50 μg/ml kanamycin with 1 ml of the overnight culture was grown at 37°C with shaking (200 rpm) until the optical density reached 0.4–0.6. CSP1 and CSP3 proteins were induced with 1mM isopropyl β-D-thiogalactoside (IPTG) for 3 h at 37°C, while CSP2 protein was induced with IPTG for 3 h at 32°C. In the three experiments, the cells were harvested by centrifugation at 5000 rpm for 10 min, resuspended in lysis buffer (20 mM sodium phosphate, 500 mM NaCl, 10 mM imidazole, pH 8.0), lysed by sonication (10 s, 5 passes) and centrifuged again at 12 000 rpm for 15 min at 4°C. Proteins from the clear supernatant and the precipitated pellet were then collected separately and analysed by SDS-PAGE. Western blot was done to attest the identity of the expressed protein. The proteins separated by SDS-PAGE were transferred to nitrocellulose blotting membranes (Pall Corporation) at 1mA/cm^2^ for 2 h using semi-dry transfer system JY-ZY3 (Beijing Junyi-Dongfang Electrophoresis Equipment CO. LTD). Blocking was done in TBST (10 mM Tris-HCl, 0.15 M NaCl, 0.05% Tween-20) overnight at 4°C. *Heliothis* CSP1b antibody was used as primary antibody at dilution of 1:1000 as described in Xuan et al. [45]. Anti-rabbit IgG labeled with HRP was used as secondary antibody at a dilution of 1:3000. Protein was detected using the HRP-DAB chromogenic substrate detection system (Tiangen) as described by the manufacturer.

SUMO-BtabCSP1, BtabCSP2 and BtabCSP3 fusion proteins were purified through Ni-NTA column and gel-filtration on a Superdax 75 column conditioned in the AKTA Purifier 10 system (GE Healthcare Biosciences). The N-terminal peptide containing the 6xHis tag and SUMO was cleaved using SUMO protease (Invitrogen) following the manufacturer’s recommendations and the cleaved protein purified using Ni-NTA column. Resulting peak protein fractions were desalted by HiTrap desalting column (GE Healthcare Biosciences) and concentrated using 3-kDa cutoff filters (Millipore). The final concentration of pure recombinant CSP1, CSP2 and CSP3 protein samples was assessed by the Bradford method using BSA as standard.

### Fluorescence binding assay

Protein-ligand binding affinity was determined for the three recombinant proteins. Emission fluorescence spectra were recorded on a Jasco FP-6500 Series fluorescence spectrofluorometer. To measure the affinity of the fluorescence ligand of 1-NPN to CSP1, CSP2 and CSP3, a 2 μM solution of protein in 50 mM Tris buffer (pH 7.4) was titrated with aliquots of 1 mM methanol solutions of ligand to a final concentration of 2–30 μM. Fluorescence was measured using an exciting wavelength of 337 nm. Emission spectrum was recorded between 350 and 500 nm. Ligand affinity was measured in competitive fluorescence binding assay using 2 μM of 1-NPN as fluorescent reporter and 2–50 μM of a competitor ligand solution. Binding constants of competitors were calculated from IC50 values using the equation: Ki = [IC50]/ (1+[1-NPN]/ K_1-NPN_), [1-NPN] and K_1-NPN_ being the free concentration of 1-NPN and the dissociation constant of the complex CSP/1-NPN, respectively [91].

### Molecular modeling of CSP1, CSP2 and CSP3

3D models for BtabCSP1, BtabCSP2 and BtabCSP3 were built using Modeller [92]. For each of these three targets, appropriate structural templates were obtained manually from the PDB after a Blast search. Seven structural templates were found in PDB (1K19, 1KX8, 1KX9, 1N8U, 1N8V, 2JNT, 2GVS). These shared pairwise sequence identities between 37.1% and 40% with BtabCSP1, between 42% and 50% with BtabCSP2 and between 45.3% and 54.1% with BtabCSP3. Two of them (1N8U, 1N8V) were in the holo form and contained a bound ligand while the others were in the apo form and did not contain a ligand (1K19, 1KX8, 1KX9, 2JNT, 2GVS). The former differed sensibly to the apo form for they have a well-identified large cavity thus showing that CSP proteins undergo structural changes upon binding to ligands. Both apo and holo forms were used for the modelling. They were aligned with the BtabCSP targets using multiple sequence alignment in the Geneious software and the alignments were used by Modeller using the standard *automodel* procedure and both *VTFM* and *MD* optimization procedures for building and refining 3D models for each of the BtabCSP. In order to take into account inherent flexibility of the protein, 100 models for each BtabCSP were built. These display structural variations namely at the side chains levels hence mimicking protein flexibility. These were used in the subsequent docking detailed below.

### Molecular docking

The binding of linoleic acid and α-pentyl-cinnamaldehyde to BtabCSP1, BtabCSP2 and BtabCSP3 was further assessed using molecular docking simulations. For each CSP, docking of the ligands were performed on the 100 models using AutodockVina 4.2 with standard docking parameters, a grid that englobed all the protein and ligands that were left completely flexible [93]. For each CSP, the most populated cluster of ligand poses across all the 100 protein models was selected as the most representative binding mode of the ligand. From these, average predicted binding energy (in kcal/mol) were calculated and corresponding average predicted K_d_ values (in μM) were derived where K_d_ = exp(ΔG/RT) with T = 300 K. In order to take into account the flexibility of the protein, this K_d_ valued was further weighted by the fraction *free*/*bound* where *free* is the amount of models among 100 where the most populated ligand pose was not observed and bound is the amount of models among 100 where the most populated ligand pose was observed. Qualitative analysis of the residues involved in the interaction with the ligands was performed using the LigPlot+ software [94].

## Supporting Information

S1 Fig Predicted secondary and tertiary structure of three *B*. *tabaci* CSPs. Hydropathy and relative flexibility plots (http://web.expasy.org/cg-bin/protscale). Bars in black indicate the position of putative flexible protein domains (A). Homology modeling using *M*. *brassicae* CSPA6 (1n8v.1.A; X-ray, 1.4 Å) as template (http://swissmodel.expasy.org) (B).
(EPS)

S2 Fig Comparison of *B*.*tabaci* CSP-encoding cDNA nucleotide sequences between B and Q. CSP2 (A), CSP3 (B). CSP1-RNA mutations are reported in Liu et al. [37].
(DOC)

S3 Fig Agarose gel electrophoretic analysis of *B*. *tabaci* CSP1-genomic DNA PCR products from B and Q individuals of *B*. *tabaci*. Qm: Q males, Qf: Q females, Bm: B males, Bf: B females. Ten individuals were tested in each category (1–10). The arrow tip indicates the occurrence of two CSP1-gDNA products in Q-biotype.
(EPS)

S4 Fig Comparison of B. *tabaci* CSP-encoding genomic DNA nucleotide sequences between B and Q. (A) CSP1, (B) CSP2 and (C) CSP3.
(DOCX)

S5 Fig Amino acid sequence alignment of *B*. *tabaci* CSP1, CSP2 and CSP3 with orthologs from other insect species. (A) CSP1, (B) CSP2 and (C) CSP3.
(DOCX)

S6 Fig Comparative *B*. *tabaci* CSP1, CSP2 and CSP3 gene expression between B and Q biotypes. Relative gene expression levels for CSP1, CSP2 and CSP3 in B and Q biotype *B*. *tabaci* males and females (reference gene = β-actin). The relative expression levels observed in Q biotype males (Qm) were used for calibration (Qm calibration value = 1). Data are means ± standard deviation (n = 9). Different letters indicate significant differences at α = 0.05 by one-way Anova.
(EPS)

S7 Fig Developmental profiling and insecticide response of related CYP, Trehalase, EcR and JHBP genes in B and Q. Relative gene expression levels for CYP6CM1, CYP6CX1, Trehalase, EcR and JHBP gene expression levels are compared between 3^rd^ instar nymphs and one day-old (1d) adults and between thiamethoxam-treated and untreated mixed adults. CYP6CM1, CYP6CX1, Trehalase, EcR and JHBP gene expression was measured in thiamethoxam-treated individuals in B and Q biotypes after 24 h exposure to a 50 μM dose of thiamethoxam (24h-50) and compared to untreated control individuals (24h-0). The relative expression levels observed at the third instar nymphal stage in Q were used for calibration (calibration value = 1). Data are means ± standard deviation (n = 9). Different letters indicate significant differences at α = 0.05 by one-way Anova.
(EPS)

S8 Fig Expression, purification and 1-NPN binding of *B*. *tabaci* CSP1, CSP2 and CSP3 proteins. (A) SDS-PAGE analysis of the SUMO BtabCSP1-, BtabCSP2- and BtabCSP3- tag protein expression in *E*. *coli*. Lane 1: Protein molecular weight marker (from top: 100, 62, 40, 30, 24, 12 Kda), Lane 2: non-induced protein, Lane 3: induced protein, Lane 4: purified protein by Ni-NTA, Lane 5: pure protein after cutting His-tag tail, Lane 6: protein immunoblot using HarmCSP1b antibody. (B) Binding of 1-NPN to BtabCSP1, BtabCSP2 and BtabCSP3. The scatchart plots (inserted ones) indicate that the binding constants were 11.66, 20.77 and 15.32 μM for CSP1, CSP2 and CSP3, respectively.
(EPS)

S9 Fig Competitive binding of different classes of chemical messengers to BtabCSP1 protein. Alcohols, aldehydes, alkanes, carboxylic acids, esters, ketones, terpenes and other chemical structures including insecticide thiamethoxam were added stepwise to a 1 μM solution of *B*. *tabaci* CSP1 protein and 1-NPN (2 μM) in 0.5–5 μl aliquots from a 10 mM stock solution of the ligand. Values of fluorescence (wavelength scan: 350–500 nM) were calculated as percent of the total fluorescence in absence of competitor and plotted against chemical ligand concentrations. (A) Alcohols, (B) Aldehydes, (C) Alkanes, (D) Carboxylic acids, (E) Esters, (F) Ketones, (G) Terpenes and (H) other types of ligands. Preferential binding is clearly shown for carboxylic linoleic acid (D).
(EPS)

S10 Fig Competitive binding of linoleic acid and α-pentyl-cinnamaldehyde to CSP2 and CSP3. (EPS)

S11 Fig Binding modes of linoleic acid to CSP1 and α-pentyl-cinnamaldehyde to CSP1, CSP2 and CSP3. An alignment of the amino acid sequences of the mature proteins (after removal of peptide signal) and the α-helical profiles for each CSP is also provided. Residue numberings in these profiles are those that are featured in the text and in the LigPlot charts in Fig 7.
(EPS)

S12 Fig Tissue-distribution of *B*. *tabaci* CSP1 in Q. One-step RT-PCR amplification of BtabCSP1 RNA using various young adult whitefly tissues. A: antennae, Ab: abdomen, Th: thorax, L: legs, H: head, Wg: wings. Actin and RNA controls are shown below. Using CSP1 primers show RNA amplicon (380 bps) ubiquitously expressed in all tissues investigated.
(EPS)

S1 Table Sense nucleotide substitutions on *B*. *tabaci* CSP2 and CSP3-RNA in B and Q. *indicates frameshift and early-stop codon mutation.
(DOC)

S2 Table Missense nucleotide substitutions on *B*. *tabaci* CSP2 and CSP3-RNA from B males, B females, Q males and Q females. (DOCX)

S3 Table Chemical structure and purity of ligands used in binding assay. (DOC)

## References

[1] De Barro PJ, Liu SS, Boykin LM, Dinsdale AB. *Bemisia tabaci*: A statement of species status. Annu Rev Entomol. 2011; 56: 1–19. 10.1146/annurev-ento-112408-085504 20690829

[2] Liu SS, De Barro PJ, Xu J, Luan JB, Zang LS, Ruan YM, et al Asymmetric mating interactions drive widespread invasion and displacement in a whitefly. Science 2007; 318: 1769–1772. 17991828 10.1126/science.1149887

[3] Brown JK, Frohlich DR, Rosell RC. The sweet potato or silverleaf whiteflies: biotype of *Bemisia tabaci* or a species complex. Annu Rev Entomol. 1995; 40: 511–534.

[4] Leshkowitz D, Gazit S, Reuveni E, Ghanim M, Czosnek H, McKenzie C, et al Whitefly (*Bemisia tabaci*) genome project: analysis of sequenced clones from egg, instar and adult (viruliferous and non-viruliferous) cDNA libraries. BMC Genomics 2006; 7: 79–98. 16608516 10.1186/1471-2164-7-79 PMC1488848

[5] Inbar M, Gerling D. Plant-mediated interactions between whiteflies, herbivores, and natural ennemies. Annu Rev Entomol. 2008; 53: 431–448. 17877454 10.1146/annurev.ento.53.032107.122456

[6] Nauen R, Stumpf N, Elbert A. Toxicological and mechanistic studies on neonicotinoid cross resistance in Q-type *Bemisia tabaci* (Hemipteran: Aleyrodidae). Pest Manag Sci. 2002; 58: 868–875. 12233176 10.1002/ps.557

[7] Nauen R, Denholm I. Resistance of insect pests to neonicotinoid insecticides: Current status and future prospects. Arch Insect Biochem Physiol. 2005; 58: 200–215. 15756698 10.1002/arch.20043

[8] Horowitz AR, Kontsedalov S, Khasdan V, Ishaaya I. Biotypes B and Q of *Bemisia tabaci* and their relevance to neonicotinoid and pyriproxyfen resistance. Arch Insect Biochem Physiol. 2005; 58: 216–225. 15756703 10.1002/arch.20044

[9] Ghanim M, Kontsedalov S. Gene expression in pyriproxyfen-resistant *Bemisia tabaci* Q biotype. Pest Manag Sci. 2007; 63: 776–783. 17569108 10.1002/ps.1410

[10] Yang NN, Xie W, Yang X, Wang SL, Wu QJ, Li RM. Transcriptomic and proteomic responses of sweetpotato whitefly, *Bemisia tabaci*, to thiamethoxam. PLOS One 2013; 8: e61820 10.1371/journal.pone.0061820 23671574 PMC3650016

[11] Picimbon JF, Leal WS. Olfactory soluble proteins of cockroaches. Insect Biochem Mol Biol. 1999; 30: 973–978.

[12] Picimbon JF, Dietrich K, Breer H, Krieger J. Chemosensory proteins of *Locusta migratoria* (Orthoptera: Acrididae). Insect Biochem Mol Biol. 2000a; 30: 233–241. 10732991 10.1016/s0965-1748(99)00121-6

[13] Picimbon JF, Dietrich K, Angeli S, Scaloni A, Krieger J, Breer H, et al Purification and molecular cloning of chemosensory proteins from *Bombyx mori*. Arch Insect Biochem Physiol. 2000b; 44: 120–129. 10897093 10.1002/1520-6327(200007)44:3<120::AID-ARCH3>3.0.CO;2-H

[14] Jacobs SP, Liggins AP, Zhou JJ, Pickett JA, Jin X, Field LM. OS-D-like genes and their expression in aphids (Hemiptera: Aphididae). Insect Mol Biol. 2005; 14: 423–432. 16033435 10.1111/j.1365-2583.2005.00573.x

[15] Zhou JJ, Vieira FG, He XL, Smadja C, Liu R, Rozas J. Genome annotation and comparative analyses of the odorant-binding proteins and chemosensory proteins in the pea aphid *Acyrthosiphon pisum*. Insect Mol Biol. 2010; 19: 113–122. 10.1111/j.1365-2583.2009.00919.x 20482644

[16] Hua JF, Zhang S, Cui JJ, Wang DJ, Wang CY, Luo JY, et al Functional characterizations of one odorant-binding protein and three chemosensory proteins from *Apolygus lucorum* (Meyer-Dur) (Hemiptera: Miridae) legs. J Insect Physiol. 2013; 59: 690–696. 10.1016/j.jinsphys.2013.04.013 23665333

[17] Nomura A, Kawasaki K, Kubo T, Natori S. Purification and localization of p10, a novel protein that increases in nymphal regenerating legs of *Periplaneta americana* (American cockroach). Int J Dev Biol. 1992; 36: 391–398. 1445782

[18] McKenna MP, Hekmat-Scafe DS, Gaines P, Carlson JR. Putative *Drosophila* pheromone-binding-proteins expressed in a subregion of the olfactory system. J Biol Chem. 1994; 269: 16340–16347. 8206941

[19] Pikielny CW, Hasan G, Rouyer F, Rosbach M. Members of a family of *Drosophila* putative odorant-binding proteins are expressed in different subsets of olfactory hairs. Neuron 1994; 12: 35–49. 7545907 10.1016/0896-6273(94)90150-3

[20] Picimbon JF. Biochemistry and evolution of CSP and OBP proteins In: Blomquist GJ., Vogt R.G., editors. Insect Pheromone Biochemistry and Molecular Biology, The Biosynthesis and Detection of Pheromones and Plant Volatiles. Elsevier Academic Press, London, San Diego; 2003, pp. 539–566.

[21] Angeli S, Ceron F, Scaloni A, Monti M, Monteforti G, Minnocci A, et al Purification, structural characterization, cloning and immunocytochemical localization of chemoreception proteins from *Schistocerca gregaria*. Eur J Biochem. 1999; 262: 745–754. 10411636 10.1046/j.1432-1327.1999.00438.x

[22] Ozaki M, Wada-Katsumata A, Fujikawa K, Iwasaki M, Yokohari F, Satoji Y, et al Ant nestmate and non-nestmate discrimination by a chemosensory sensillum. Science 2005; 309: 311–314. 15947139 10.1126/science.1105244

[23] Maleszka J, Forêt S, Saint R, Maleszka R. RNAi-induced phenotypes suggest a novel role for a chemosensory protein CSP5 in the development of embryonic integument in the honeybee (*Apis mellifera*). Dev Genes Evol. 2007; 217: 189–196. 17216269 10.1007/s00427-006-0127-y

[24] Picimbon JF, Dietrich K, Krieger J, Breer H. Identity and expression pattern of chemosensory proteins in *Heliothis virescens* (Lepidoptera, Noctuidae). Insect Biochem Mol Biol. 2001; 31: 1173–1181. 11583930 10.1016/s0965-1748(01)00063-7

[25] Wanner KW, Isman MB, Feng Q, Plettner E, Theilmann DA. Developmental expression patterns of four chemosensory protein genes from the Eastern spruce budworm, *Choristoneura fumiferana*. Insect Mol Biol. 2005; 14: 289–300. 15926898 10.1111/j.1365-2583.2005.00559.x

[26] Forêt S, Wanner KW, Maleszka R. Chemosensory proteins in the honeybee: Insights from the annotated genome, comparative analysis and expression profiling. Insect Biochem Mol Biol. 2007; 37: 19–28. 17175443 10.1016/j.ibmb.2006.09.009

[27] Gong DP, Zhang H, Zhao P, Lin Y, Xia QY, Xiang ZH. Identification and expression pattern of the chemosensory protein gene family in the silkworm, *Bombyx mori*. Insect Biochem Mol Biol. 2007; 37: 266–277. 17296501 10.1016/j.ibmb.2006.11.012

[28] Xu YL, He P, Zhang L, Fang SQ, Dong SL, Zhang YJ, et al Large-scale identification of odorant-binding proteins and chemosensory proteins from expressed sequence tags in insects. BMC Genomics 2009; 10: 632 10.1186/1471-2164-10-632 20034407 PMC2808328

[29] Liu XL, Luo Q, Zhong GH, Rizwan-ul-Haq M, Hu M. Molecular characterization and expression pattern of four chemosensory proteins from diamondback moth, *Plutella xylostella* (Lepidoptera: Plutellidae). J Biochem. 2010; 148: 189–200. 10.1093/jb/mvq050 20495011

[30] Zhou XH, Ban LP, Iovinella I, Zhao LJ, Gao Q, Felicioli A, et al Diversity, abundance and sex-specific expression of chemosensory proteins in the reproductive organs of the locust *Locusta migratoria manilensis*. Biol Chem. 2012; 394: 43–54. 10.1515/hsz-2012-0114 23096575

[31] Zhang YN, Jin JY, Jin R, Xia YH, Zhou JJ, Deng JY, et al Differential expression patterns in chemosensory and non-chemosensory tissues of putative chemosensory genes identified by transcriptome analysis of insect pest the purple stem borer *Sesamia inferens* (Walker). PLOS One 2013; 8: e69715 10.1371/journal.pone.0069715 23894529 PMC3722147

[32] Picimbon JF. Renaming *Bombyx mori* Chemosensory Proteins. Int J Bioorg Chem Mol Biol. 2014a; 2: 201.

[33] Bos JI, Prince D, Pitino M, Maffei ME, Win J, Hogenhout SA. A functional genomics approach identifies candidate effectors from the aphid species *Myzus persicae* (Green Peach Aphid). PLOS Genet. 2010; 6: e1001216 10.1371/journal.pgen.1001216 21124944 PMC2987835

[34] Liu YL, Guo H, Huang LQ, Pelosi P, Wang CZ. Unique function of a chemosensory protein in the proboscis of two *Helicoverpa* species. J Exp Biol. 2014; 217: 1821–1826. 10.1242/jeb.102020 24625642

[35] Sabatier L, Jouanguy E, Dostert C, Zachary D, Dimarcq JL, Bulet P, et al Pherokine-2 and -3: Two *Drosophila* molecules related to pheromone/odor-binding proteins induced by viral and bacterial infections. Eur J Biol. 2003; 270: 3398–3407. 10.1046/j.1432-1033.2003.03725.x 12899697

[36] Xuan N, Guo X, Xie HY, Lou QN, Bo LX, Liu GX, et al Increased expression of CSP and CYP genes in adult silkworm females exposed to avermectins. Insect Sci. 2015; 22: 203–219. 10.1111/1744-7917.12116 24677614

[37] Liu GX, Xuan N, Chu D, Xie HY, Fan ZX, Bi YP, et al Biotype expression and insecticide response of *Bemisia tabaci* chemosensory protein-1. Arch Insect Biochem Physiol. 2014; 85: 137–151. 10.1002/arch.21148 24478049

[38] Liu GX, Ma HM, Xie HY, Xuan N, Picimbon JF. Sequence variation of *Bemisia tabaci* Chemosensory protein 2 in cryptic species B and Q: new DNA markers for whitefly recognition. Gene 2016; 576: 284–291. 10.1016/j.gene.2015.10.036 26481237

[39] Wanner KW, Willis LG, Theilmann DA, Isman MB, Feng Q, Plettner E. Analysis of the insect os-d-like gene family. J Chem Ecol. 2004; 30: 889–911. 15274438 10.1023/b:joec.0000028457.51147.d4

[40] Ozaki K, Utoguchi A, Yamada A, Yoshikawa H. Identification and genomic structure of chemosensory protein (CSP) and odorant binding protein (OBP) genes expressed in foreleg tarsi of the swallowtail butterfly *Papilio xuthus*. Insect Biochem Mol Biol. 2008; 38: 969–976. 10.1016/j.ibmb.2008.07.010 18771731

[41] Kulmuni J, Wurm Y, Pamilo P. Comparative genomics of chemosensory protein genes reveals rapid evolution and positive selection in ant-specific duplicates. Heredity 2013; 110: 538–547. 10.1038/hdy.2012.122 23403962 PMC3656642

[42] Wang R, Zhang X, Li H, Guo X, Luo C. Identification and expression profiling of five chemosensory protein genes in the whitefly MED, *Bemisia tabaci*. J Asia-Pacific Entomol. 2016; 19: 195–201.

[43] Ma DY, Gorman K, Devine G, Luo W, Denholm I. The biotype and insecticide-resistance status of whiteflies, *Bemisia tabaci* (Hemiptera: Aleyrodidae), invading cropping systems in Xinjiang Uygur Autonomous Region, northwestern China. Crop Prot. 2007; 26: 612–617.

[44] Luo C, Jones CM, Devine G, Zhang F, Denholm I, Gorman K. Insecticide resistance in *Bemisia tabaci* biotype Q (Hemiptera: Aleyrodidae) from China. Crop Prot. 2010; 29: 429–434.

[45] Xuan N, Bu X, Liu YY, Yang X, Liu GX, Fan ZX, et al Molecular evidence of RNA editing in *Bombyx* chemosensory protein family. PLOS One 2014; 9: e86932 10.1371/journal.pone.0086932 24551045 PMC3923736

[46] Picimbon JF. RNA mutations: source of life. GNT 2014b; 4: 112.

[47] Tay WT, Evans GA, Boykin LM, De Barro PJ. Will the real *Bemisia tabaci* please stand up? PLOS One 2012; 7: e50550 10.1371/journal.pone.0050550 23209778 PMC3509048

[48] Boykin LM, Bell CD, Evans G, Small I, De Barro PJ. Is agriculture driving the diversification of the *Bemisia tabaci* species complex (Hemiptera: Sternorrhyncha: Aleyrodidae)? Dating, diversification and biogeographic evidence revealed. BMC Evol Biol. 2013; 13: 228 10.1186/1471-2148-13-228 24138220 PMC3853546

[49] Picimbon JF. RNA mutations in the moth pheromone gland. RNA Dis. 2014c; 1: 1–6.

[50] Briand L, Swasdipan N, Nespoulos C, Bézirard V, Blon F, Huet JC, et al Characterization of a chemosensory protein (ASP3c) from honeybee (*Apis mellifera* L.) as a brood pheromone carrier. Eur J Biochem. 2002; 269: 4586–4596. 12230571 10.1046/j.1432-1033.2002.03156.x

[51] Calvello M, Brandazza A, Navarrini A, Dani FR, Turillazzi S, Felicioli A, et al Expression of odorant-binding proteins and chemosensory proteins in some hymenoptera. Insect Biochem Mol Biol. 2005; 35: 297–307. 15763466 10.1016/j.ibmb.2005.01.002

[52] Gätschenberger H, Gimple O, Tautz J, Beier H. Honey bee drones maintain humoral immune competence throughout all life stages in the absence of vitellogenin production. J Exp Biol. 2012; 215: 1313–1322. 10.1242/jeb.065276 22442369

[53] Guo W, Wang XH, Ma ZY, Xue L, Han JY, Yu D, et al CSP and Takeout genes modulate the switch between attraction and repulsion during behavioral phase change in the migratory locust. PLOS Genet. 2011; 7: e1001291 10.1371/journal.pgen.1001291 21304893 PMC3033386

[54] Ghanim M, Dombrovsky A, Raccah B, Sherman A. A microarray approach identifies ANT, OS-D and takeout-like genes as differentially regulated in alate and apterous morphs of the green peach aphid *Myzus persicae* (Sulzer). Insect Biochem Mol Biol. 2006; 36: 857–868. 17046599 10.1016/j.ibmb.2006.08.007

[55] Liu R, He X, Lehane S, Lehane M, Hertz-Fowler C, Berriman M, et al Expression of chemosensory proteins in the tsetse fly *Glossina morsitans morsitans* is related to female host-seeking behaviour. Insect Mol Biol. 2012; 21: 41–48. 10.1111/j.1365-2583.2011.01114.x 22074189 PMC3664020

[56] Gu SH, Wang SY, Zhang XY, Ji P, Liu JT, Wang GR, et al Functional characterization of chemosensory proteins of the alfalfa plant bug *Adelphocoris lineolatus* indicates their involvement in host recognition. PLOS One 2012; 7: e42871 10.1371/journal.pone.0042871 22900060 PMC3416781

[57] Buckner JS, Hagen MM, Nelson RR. The composition of the cuticular lipids from nymphs and exuviae of the silverleaf whitefly, *Bemisia argentifolii*. Comp Biochem Physiol B: Biochem Mol Biol. 1999; 124: 201–207.

[58] Mahadav A, Gerling D, Gottlieb Y, Czosnek H, Ghanim M. Parasitization by the wasp *Eretmocerus mundus* induces transcription of genes related to immune response and symbiotic bacteria proliferation in the whitefly *Bemisia tabaci*. BMC Genomics 2008; 9: 342 10.1186/1471-2164-9-342 18638407 PMC2488360

[59] Su YL, Li JM, Li M, Luan JB, Ye XD, Wang XW, et al Transcriptomic analysis of the salivary glands of an invasive whitefly. PLOS One 2012; 7: e39303 10.1371/journal.pone.0039303 22745728 PMC3379992

[60] Karunker I, Benting J, Lueke B, Ponge T, Nauen R, Roditakis E, et al Over-expression of cytochrome P450 CYP6CM1 is associated with high resistance to imidacloprid in the B and Q biotypes of *Bemisia tabaci* (Hemiptera: Aleyrodidae). Insect Biochem Mol Biol. 2008; 38: 634–644. 10.1016/j.ibmb.2008.03.008 18510975

[61] James RR, Xu J. Mechanisms by which pesticides affect insect immunity. J Invertebr Pathol. 2012; 109: 175–182. 10.1016/j.jip.2011.12.005 22206912

[62] Cutler C. Insects, insecticides and hormesis: evidence and considerations for study. Dose Resp. 2013; 11: 154–177. 10.2203/dose-response.12-008.Cutler PMC3682195 23930099

[63] Zhu F, Li T, Zhang L, Liu N. Co-up-regulation of three P450 genes in response to permethrin exposure in permethrin resistant house flies, *Musca domestica*. BMC Physiol. 2008; 8: 18 10.1186/1472-6793-8-18 18817570 PMC2567968

[64] Xia J, Zhang CR, Zhang S, Li FF, Feng MG, Wang XW, et al Analysis of whitefly transcriptional responses to *Beauveria bassiana* infection reveals new insights into insect-fungus interactions. PLOS ONE 2013; 8: e68185 10.1371/journal.pone.0068185 23861870 PMC3702578

[65] Daniels M, Bale JS, Newbury HJ, Lind RJ, Pritchard J. A sublethal dose of thiamethoxam causes a reduction in xylem feeding by the bird cherry-oat aphid (*Rhopalosiphum padi*), which is associated with dehydration and reduced performance. J Insect Physiol. 2009; 55: 758–765. 10.1016/j.jinsphys.2009.03.002 19482292

[66] He Y, Zhao J, Zheng Y, Weng Q, Biondi A, Desneux N, et al Assessment of potential sublethal effects of various insecticides on key biological traits of the tobacco whitefly, *Bemisia tabaci*. Int J Biol Sci. 2013; 9: 246–255. 10.7150/ijbs.5762 23494876 PMC3596710

[67] Tufail M, Nagaba Y, Elgendy AM, Takeda M. Regulation of vitellogenin genes in insects. Entomol Sci. 2014; 17: 269–282.

[68] Wang XW, Luan JB, Li JM, Bao YY, Zhang CX, Liu SS. *De novo* characterization of a whitefly transcriptome and analysis of its gene expression during development. BMC Genomics 2010; 11: 400 10.1186/1471-2164-11-400 20573269 PMC2898760

[69] Iovinella I, Bozza F, Caputo B, Della Torre A, Pelosi P. Ligand-binding study of *Anopheles gambiae* chemosensory proteins. Chem Senses 2013; 4: 3–11. 10.1093/chemse/bjt012 23599217

[70] Campanacci V, Krieger J, Bette S, Sturgis JN, Lartigue A, Cambillau C, et al Revisiting the specificity of *Mamestra brassicae* and *Antheraea polyphemus* pheromone-binding proteins with a fluorescence binding assay. J Biol Chem. 2001; 276: 20078–20084. 11274212 10.1074/jbc.M100713200

[71] Ishida Y, Tsuchiya W, Fujii T, Fujimoto Z, Miyazawa M, Ishibashi J, et al Niemann-Pick type C2 protein mediating chemical communication in the worker ant. Proc Natl Acad Sci USA 2014; 111: 3847–3852. 10.1073/pnas.1323928111 24567405 PMC3956204

[72] Lartigue A, Campanacci V, Roussel A, Larsson AM, Jones TA, Tegoni M, et al X-ray structure and ligand binding study of a moth chemosensory protein. J Biol Chem. 2002; 277: 32094–32098. 12068017 10.1074/jbc.M204371200

[73] Tomaselli S, Crescenzi O, Sanfelice D, Ab E, Wechselberger R, Angeli S, et al Solution structure of a chemosensory protein from the desert locust *Schistocerca gregaria*. Biochemistry 2006; 45: 1606–1613. 10.1021/bi060998w 16939212

[74] Jansen S, Chmelik J, Zídek L, Padrta P, Novak P, Zdráhal Z, et al Structure of *Bombyx mori* Chemosensory Protein 1 in solution. Arch Insect Biochem Physiol. 2007; 66: 135–145. 17966128 10.1002/arch.20205

[75] Pelosi P, Zhou JJ, Ban LP, Calvello M. Soluble proteins in insect chemical communication. Cell Mol Life Sci. 2006; 63: 1658–1676. 16786224 10.1007/s00018-005-5607-0 PMC11136032

[76] Yi X, Zhao H, Dong X, Wang P, Hu M, Zhong G. BdorCSP2 is important for antifeed and oviposition-deterring activities induced by Rhodojaponin-III against *Bactrocera dorsalis*. PLOS One 2013; 8: e77295 10.1371/journal.pone.0077295 24155937 PMC3796470

[77] Blomquist GJ, Dwyer LA, Chu AJ, Ryan RO, de Renobales M. Biosynthesis of linoleic acid in a termite, cockroach and cricket. Insect Biochem. 1982; 12: 349–353.

[78] Tillman JA, Seybold SJ, Jurenka RA, Blomquist GJ. Insect pheromones-an overview of biosynthesis and endocrine regulation. Insect Biochem Mol Biol. 1999; 29: 481–514. 10406089 10.1016/s0965-1748(99)00016-8

[79] Blaul B, Steinbauer R, Merkl P, Merkl R, Tschochner H, Ruther J. Oleic acid is a precursor of linoleic acid and the male sex pheromone in *Nasonia vitripennis*. Insect Biochem Mol Biol. 2014; 51C: 33–40. 10.1016/j.ibmb.2014.05.007 24874439

[80] Liu B, Jiang G, Zhang Y, Li J, Li X, Yue J, et al Analysis of transcriptome differences between resistant and susceptible strains of the citrus red mite *Panonychus citri* (Acari: Tetranychidae). PLOS One 2011; 6: e28516 10.1371/journal.pone.0028516 22162774 PMC3230605

[81] Lin QS, Jin FL, Hu ZD, Chen HY, Yin F, Li Z. Transcriptome analysis of chlorantraniliprole resistance development in the diamondback moth *Plutella xylostella*. PLOS One 2013; 8: e72314 10.1371/journal.pone.0072314 23977278 PMC3748044

[82] Hosoda K, Shimomura H, Hayashi S, Yokota K, Hirai Y. Steroid hormones as bacterial agents to *Helicobacter pylori*. FEMS Microbiol Lett. 2011; 318: 68–75. 10.1111/j.1574-6968.2011.02239.x 21306429

[83] Amgalanbaatar A, Shimomura H, Hosoda K, Hayashi S, Yokota K, Hirai Y. Antibacterial activity of a novel synthetic progesterone species carrying a linoleic acid molecule against *Helicobacter pylori* and the hormonal effect of its steroid on a murine macrophage-like cell line. J. Steroid Biochem Mol Biol. 2014; 140: 17–25. 10.1016/j.jsbmb.2013.10.023 24189541

[84] Huang Y, Ho SH. Toxicity and antifeedant activities of cinnamaldehyde against the grain storage insects, *Tribolium castaneum* (Herbst) and *Sitophilus zeamais* Motsch. J Stored Prod Res. 1998; 34: 11–17.

[85] George DR, Sparagano OAE, Port G, Okello E, Shiel RS, Guy JH. Toxicity of plant essential oils to different life stages of the poultry red mite, *Dermanyssus gallinae*, and non-target invertebrates. Med Vet Entomol. 2010; 24: 9–15. 10.1111/j.1365-2915.2009.00856.x 20377726

[86] Wang Z, Kim HK, Tao W, Wang M, Ahn YJ. Contact and fumigant toxicity of cinnamaldehyde and cinnamic acid and related compounds to *Dermatophagoides farina* and *Dermatophagoides pteronyssinus* (Acari: Pyroglyphidae). J Med Entomol. 2011; 48: 366–371. 21485375 10.1603/me10127

[87] Chu D, Jiang T, Liu GX, Jiang DF, Tao YL, Fan ZX, et al Biotype status and distribution of *Bemisia tabaci* (Hemiptera: Aleyrodidae) in Shandong Province of China based on mitochondrial DNA markers. Mol Ecol Evol. 2007; 36: 1290–1295. 10.1603/0046-225x(2007)36[1290:bsadob]2.0.co;2 18284755

[88] Daniele S, Michalak I. raxmlGUI: a graphical front-end for RAxML. Org Div Evol. 2012; 12: 335–337.

[89] Livak KJ, Schmittgen TD. Analysis of relative gene expression data using real-time quantitative PCR and the 2(-Delta Delta C(T)) method. Methods 2001; 25: 402–408. 11846609 10.1006/meth.2001.1262

[90] Rauch N, Nauen R. Identification of biochemical markers linked to neonicotinoid cross resistance in *Bemisia tabaci* (Hemiptera: Aleyrodidae). Arch Insect Biochem Physiol. 2003; 54: 165–176. 14635178 10.1002/arch.10114

[91] Ban LP, Scaloni A, Brandazza A, Angeli S, Zhang L, Yan Y, et al Chemosensory proteins of *Locusta migratoria*. Insect Mol Biol. 2003; 12: 125–134. 12653934 10.1046/j.1365-2583.2003.00394.x

[92] Sali A, Blundell TL. Comparative protein modelling by satisfaction of spatial restraints. J Mol Biol. 1993; 234: 779–815. 8254673 10.1006/jmbi.1993.1626

[93] Trott O, Olson AJ. AutoDock Vina: improving the speed and accuracy of docking with a new scoring function, efficient optimization and multithreading. J Comp Chem. 2010; 31: 455–461. 19499576 10.1002/jcc.21334 PMC3041641

[94] Laskowski RA, Swindells MB. LigPlot+: multiple ligand-protein interaction diagrams for drug discovery. J Chem Inf Model. 2011; 51: 2778–2786. 10.1021/ci200227u 21919503

