# Supplementary material for: Biotype Characterization, Developmental Profiling, Insecticide Response and Binding Property of Bemisia tabaci Chemosensory Proteins: Role of CSP in Insect Defense
Source: PLoS One. 2016 May 11;11(5):e0154706. doi: 10.1371/journal.pone.0154706 (PMC4864240; doi:10.1371/journal.pone.0154706)
Supplement: S2 Fig — CSP2 (A), CSP3 (B). CSP1-RNA mutations are reported in Liu et al. [37]. (DOC) [file pone.0154706.s002.doc]

A.

B.
