# Supplementary material for: Biotype Characterization, Developmental Profiling, Insecticide Response and Binding Property of Bemisia tabaci Chemosensory Proteins: Role of CSP in Insect Defense
Source: PLoS One. 2016 May 11;11(5):e0154706. doi: 10.1371/journal.pone.0154706 (PMC4864240; doi:10.1371/journal.pone.0154706)
Supplement: S5 Fig — (A) CSP1, (B) CSP2 and (C) CSP3. (DOCX) [file pone.0154706.s005.docx]

A.

B．

C.
