# Supplementary material for: Biotype Characterization, Developmental Profiling, Insecticide Response and Binding Property of Bemisia tabaci Chemosensory Proteins: Role of CSP in Insect Defense
Source: PLoS One. 2016 May 11;11(5):e0154706. doi: 10.1371/journal.pone.0154706 (PMC4864240; doi:10.1371/journal.pone.0154706)
Supplement: S1 Table — *indicates frameshift and early-stop codon mutation. (DOC) [file pone.0154706.s013.doc]

| **Biotype** | **Site** | **Nucleotide change** | **Frequency** | **Conserved amino acid (Y/N)** | **Accession**  **Number** |
| --- | --- | --- | --- | --- | --- |
| *BtabCSP2* |  |  |  |  |  |
| Q | 45 | C→T | 1/5 | N | KM078672 |
|  | 113 | T deletion* | 1/5 | Y* | KM078671 |
|  | 165 | C→T | 1/5 | N | KM078674 |
|  | 231 | C→A | 2/5 | N | KM078671, KM078675 |
|  | 240 | A→G | 1/5 | N | KM078671 |
|  | 258 | C→A | 2/5 | N | KM078671, KM078675 |
|  | 264 | A→C | 2/5 | N | KM078671, KM078675 |
|  | 267 | G→A | 2/5 | N | KM078671, KM078675 |
|  | 318 | C→T | 1/5 | N | KM078671 |
| B | 123 | G→A | 5/5 | N | KM078676-79 |
|  | 189 | A→G | 1/5 | N | KM078676 |
|  | 231 | C→A | 5/5 | N | KM078676-79 |
|  | 240 | A→T | 5/5 | N | KM078676-79 |
|  | 288 | G→A | 5/5 | N | KM078676-79 |
|  | 318 | C→T | 5/5 | N | KM078676-79 |
|  | 321 | C→T | 5/5 | N | KM078676-79 |
| *BtabCSP3* |  |  |  |  |  |
| B | 369 | C→T | 2/2 | N | KM078696-97 |
