# Supplementary material for: Biotype Characterization, Developmental Profiling, Insecticide Response and Binding Property of Bemisia tabaci Chemosensory Proteins: Role of CSP in Insect Defense
Source: PLoS One. 2016 May 11;11(5):e0154706. doi: 10.1371/journal.pone.0154706 (PMC4864240; doi:10.1371/journal.pone.0154706)
Supplement: S2 Table — (DOCX) [file pone.0154706.s014.docx]

| **Clone**  **number** | **Nucleotide**  **motif** | **Amino acid motif** | **Location**  **(bp)** | **Accession number** |
| --- | --- | --- | --- | --- |
| *BtabCSP2* |  |  |  |  |
| I73-Qmale1 | ATTTTGGGC | DLG | 38 | KM078671 |
|  | ATTT-GGGC | DWA |  |  |
| I80-Bfemale2 | GGCAGCAAA | GSG | 40 | KM078677 |
|  | GGCGGCAAA | GGK |  |  |
| *BtabCSP3* |  |  |  |  |
| I62-Qfemale1 | ACGTAC | ST | 31 | KM078694 |
|  | ACGAAGGAAAGTACGTAC | SKESTT |  |  |
