# Supplementary material for: Biotype Characterization, Developmental Profiling, Insecticide Response and Binding Property of Bemisia tabaci Chemosensory Proteins: Role of CSP in Insect Defense
Source: PLoS One. 2016 May 11;11(5):e0154706. doi: 10.1371/journal.pone.0154706 (PMC4864240; doi:10.1371/journal.pone.0154706)
Supplement: S3 Table — (DOC) [file pone.0154706.s015.doc]

| **Ligand name** | **Stuctural formula** | **Purity** | **Ligand name** | **Stuctural formula** | **Purity** |
| --- | --- | --- | --- | --- | --- |
| ***Alcohols*** |  |  | 6-Methyl-5-hepten-2-one | 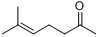 | 99% |
| Trans-2-hexen-1-ol | 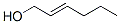 | 96% | 2-Pentadecanone | 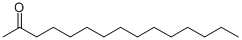 | >98% |
| Linalool | 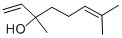 | 97% | β-Ionone | 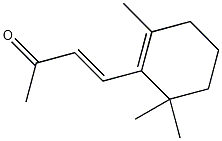 | 96% |
| 1-Hexanol | 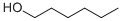 | 99% | 2,4′-Dimethylacetophenone | 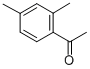 | 96% |
| Geraniol | 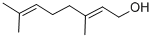 | 98% | ***Carboxylic acids*** |  |  |
| Z3-Hexen-1-ol | 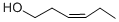 | 98% | Octanoic acid | 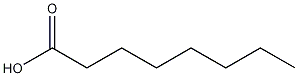 | >98% |
| (-)-Carveol | 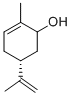 | 97% | Decanoic acid | 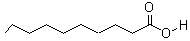 | >98% |
| 2-Ethyl-hexanol | 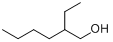 | >99% | Linoleic acid | 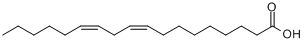 | >99% |
| 3-Hexanol | 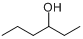 | 97% | Cinnamic acid | 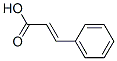 | 97% |
| α-Terpineol | 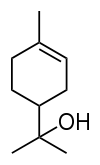 | 90% | Lauric acid | 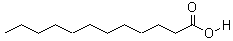 | >98% |
| (+/-)Nerolidol | 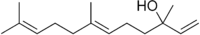 | 98% | ***Esters*** |  |  |
| Trans,trans-Farnesol | 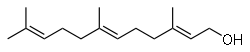 | 95% | Hexyl acetate | [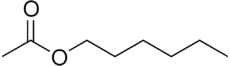](https://en.wikipedia.org/wiki/File:Hexyl_acetate.png) | >99% |
| 1-Heptanol | 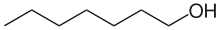 | 98% | Amyl acetate | [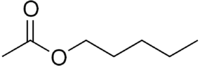](https://en.wikipedia.org/wiki/File:Amyl_acetate.png) | 99% |
| ***Aldehydes*** |  |  | Methyl salicylate | [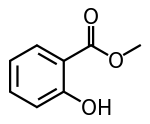](https://en.wikipedia.org/wiki/File:Methyl_salicylate.svg) | >99% |
| Heptanal | 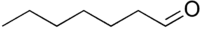 | 95% | (Z)-3-Hexenyl acetate | 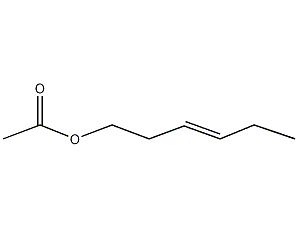 | >98% |
| Trans-2-hexenal | 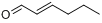 | 98% | (E)-2-Hexenyl butyrate | 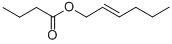 | >96% |
| α-pentyl-  cinnamaldehyde | 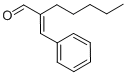 | 97% | ***Terpenes*** |  |  |
| Valeraldehyde | 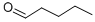 | 97% | Caryopyllene | 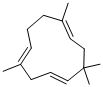 | >96% |
| Nonanal | 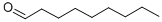 | 95% | α-Pinene | 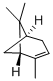 | 98% |
| Benzaldehyde | [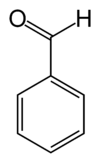](https://en.wikipedia.org/wiki/File:Benzaldehyde.png) | >99% | β-Pinene | 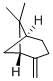 | 99% |
| n-Hexaldehyde | 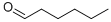 | 98% | Myrcene | 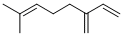 | analytical standard |
| Octanal | 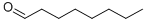 | 99% | 3-Carene | [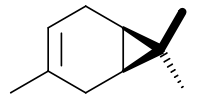](https://en.wikipedia.org/wiki/File:3-Caren.svg) | >90% |
| Dodecyl aldehyde | 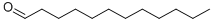 | >95% | R(+)-Limonene | 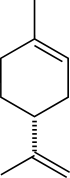 | 97% |
| ***Alkanes*** |  |  | S(-)-Limonene | 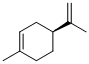 | 96% |
| n-Undecane | 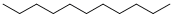 | >99% | Ocimene | 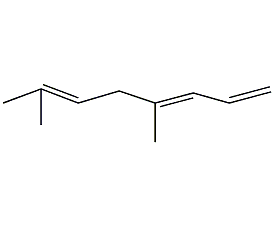 | >90% |
| n-Tridecane | 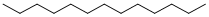 | >99% | α-Terpinene | 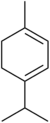 | >95% |
| n-Pentadecane | 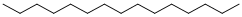 | >98% | E-β-Farnesene | 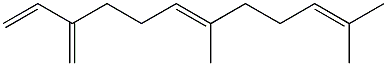 | analytical standard |
| n-Hexane | 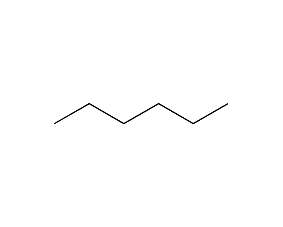 | 95% | ***Others*** |  |  |
| n-Heptane | 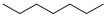 | 95% | Eugenol | 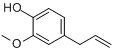 | 99% |
| ***Ketones*** |  |  | 1,8-Cineole | 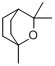 | reference standard |
| 2-Heptanone | 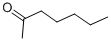 | 98% | m-Xylene | 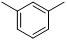 | 99% |
| (1S)-(-)-Camphor | 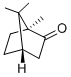 | 99% | Thiamethoxam | 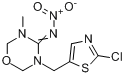 | 25% |
|  |  |  |  |  |  |
